# Critical roles of Dpb3-Dpb4 sub-complex of DNA polymerase epsilon in DNA replication, genome stability, and pathogenesis of *Candida albicans*

**DOI:** 10.1128/mbio.01227-24

**Published:** 2024-08-29

**Authors:** Bhabasha Gyanadeep Utkalaja, Shraddheya Kumar Patel, Satya Ranjan Sahu, Abinash Dutta, Narottam Acharya

**Affiliations:** 1 Laboratory of Genomic Instability and Diseases, Department of Infectious Disease Biology, Institute of Life Sciences, Bhubaneswar, India; 2 Regional Center of Biotechnology, Faridabad, India; Institut Pasteur, Paris, France; Polish Academy of Sciences, Warsaw, Poland

**Keywords:** *Candida albicans*, DNA replication, DNA polymerase, whole genome sequencing, systemic candidiasis, virulence

## Abstract

DNA polymerase ε (Polε) is an essential replicative polymerase consisting of Pol2, Dpb2, Dpb3, and Dpb4 subunits and has not been explored in the pathogenic yeast *Candida albicans. C. albicans* is accountable for >40% of deaths due to systemic candidiasis per year worldwide. Genome plasticity is one of the adaptive mechanisms associated with virulence, and as it is associated with DNA polymerase function, this study explored the role of Polε in genome stability and pathogenesis of *C. albicans. POL2* and *DPB2* are haploinsufficient, but *DPB3* and *DPB4* are dispensable for cell survival in diploid *C. albicans*. However, unlike in *Saccharomyces cerevisiae*, loss of any or both of the nonessential subunits or defective interaction between the two resulted in slow growth and temperature-sensitive phenotypes. Knockout strains of *C. albicans (dpb3*ΔΔ and *dpb4*ΔΔ and *dpb3*ΔΔ*dpb4*ΔΔ) also exhibited sensitivity to genotoxic agents and delayed cell cycle progression. Reduced processive DNA synthesis and increased rate of mutagenesis were observed in *dpb3* and *dpb4* null strains. Whole-genome sequencing further confirmed the accumulation of indels and SNPs majorly in the intergenic repeat regions of the chromosomes of *dpb3*ΔΔ*dpb4*ΔΔ. Polε-defective strains were constitutively filamentous and non-pathogenic in mice models of systemic candidiasis. Altogether, this study showed that the function of the Dpb3-Dpb4 subcomplex is critical for fungal morphogenesis and virulence besides its role as a structural component of Polε in DNA replication and genome stability; thus, their interacting interface may be targeted to develop antifungal drugs.

**IMPORTANCE:**

This study explored the role of DNA polymerase epsilon, especially its non-essential structural subunits in *Candida albicans* biology. Apart from their role in DNA replication and genome stability, the Dpb3-Dpb4 subcomplex regulates morphological switching and virulence. Since the defective strain is locked in filamentous form and is avirulent, the complex may be targeted for anti-fungal drug development.

## INTRODUCTION

In a cell, the replisome, a complex of several proteins with diverged functions, carries out DNA replication during the S phase of the cell cycle (1). In eukaryotic replisomes, DNA synthesis is coordinated by three essential replicative DNA polymerases Polα, Polδ, and Polε (2). While Polα initiates RNA-DNA primer synthesis, extensive genetic analyses in *Saccharomyces cerevisiae* suggest that Polε is involved in only leading strand DNA synthesis, and Polδ synthesizes both lagging and leading strands of DNA (2–4). Polε interacts with the CMG helicase complex and PCNA for its processive DNA synthesis, whereas Polδ interacts with PCNA alone as its processivity factor (5–7). Recently, we found that unlike PCNA, the yeast 9-1-1 complex (Rad17-Mec3-Ddc1), the other DNA clamp, binds and enhances the catalytic activity of Polε without affecting processivity (7). In addition, Polε possesses certain structural elements that are absent in Polδ, making Polε intrinsically a highly processive enzyme (8, 9). *S. cerevisiae* Polδ consists of a catalytic Pol3 and two accessory Pol31 and Pol32 subunits, whereas Polε is a holoenzyme of four subunits Pol2, Dpb2, Dpb3, and Dpb4 (10). Pol2 is the largest subunit possessing the catalytic centers for the polymerase and exonuclease domains. Interestingly, Pol2 is essential for cell survival not because of its catalytic function but rather for its structural C-terminal domain that is involved in interaction with CMG helicase, which is required for the initiation of DNA replication (11–13). However, elevated dNTP concentrations and mutation rate were observed in a *S. cerevisiae* strain defective in both polymerase and exonuclease activities in Pol2 (*pol2-16*) compared with in wild type (14). Polε also serves as a sensor of DNA replication that functions in the activation of the S phase checkpoint (15). *DPB2* is an essential structural gene in yeast, and the protein gets phosphorylated by Cdc28 in a cell cycle-dependent manner (16). Dpb2 binds to the Zn-finger containing (CysA and CysB) C- terminal domain of Pol2 via its phosphodiesterase domain (PDE) (17), and the strength of interaction between Pol2 and Dpb2 determines the fidelity of Polε (18). Although *DPB3* and *DPB4* genes are dispensable for survival in yeasts, biochemical analyses suggested that Dpb3 and Dpb4 subunits are important for the interaction between Polε and DNA substrate to increase processive DNA synthesis (19). A mooring helix (V1270–S1308; 38 residues) in the ScPol2 located exactly at the junction of N-terminal catalytic and C-terminal structural domains tethers Dpb3–Dpb4 subcomplex (17). It was reported that the deletion of *DPB3* and *DPB4* elevates spontaneous frameshift and base substitution rates *in vivo* (20). Altogether, both essential and nonessential subunits are involved in the structural integrity of Polε to contribute to accurate chromosomal DNA replication. Both Dpb3 and Dpb4 possess a histone fold-like (HF) domain through which they heterodimerize to form a complex similar to the H2A-H2B histone dimer (21). Various genetic studies suggested a role for Dpb3 and Dpb4 in heterochromatin maintenance and the *dpb3*Δ and *dpb4*Δ cells show a defect in parental H3–H4 transfer leading to compromise epigenetic inheritance (21, 22). In addition, Dpb4 is also a component of the ISW2 chromatin-remodeling complex (23). In the ISW2 complex, Dpb4 interacts with another histone fold containing protein Dls1, a Dpb3 paralog. In-depth analysis found that the Dls1–Dpb4 dimer binds the double-strand break (DSB) ends and facilitates loading of the full ISW2 complex that further facilitates MRX recruitment and DSB resection by clearing nucleosomes from the DSB ends (24). Dpb3–Dpb4 complex also takes part in DSB by recruiting Rad9 and inducing its binding to histone H3 (24).

*Candida albicans* is a commensal fungus that inflicts lethal bloodstream infections in individuals due to immunosuppression and gut dysbiosis. Despite existing antifungal arsenals, the mortality rate of systemic candidiasis caused by *C. albicans* remains >40% (25). Moreover, a safe and effective antifungal vaccine is yet to be approved for human use. Therefore, the identification of new drug targets and potential vaccine candidates is of paramount importance (26–28). *C. albicans* grow on several niches and adapt to adverse conditions in multiple ways like altering morphology, genomic plasticity, biofilm formation, etc.. Alteration in gene copy number, ploidy variation, karyotypic changes, base substitutions, and loss of heterozygosity are frequently observed in clinical isolates of *C. albicans* (29, 30). Such genomic diversity is also associated with a varied degree of virulence and drug resistance; however, the causative factors/mechanisms are not completely deciphered. The activities of replicative DNA polymerases are crucial for the stabilization and function of a genome, yet have not been explored in pathogenic *C. albicans*. Recently, we have reported functional analyses of the Pol32 subunit of Polδ in *C. albicans* (31); here, we explored the role of Polε in DNA replication, genome stability, and fungal pathogenesis. We found that the deletion of *DPB3* and *DPB4* strongly affects cell morphology and viability in mice with consequences on pathogenesis; thus, these subunits have additional functions in regulating morphogenesis and virulence of *C. albicans* apart from their roles as in *S. cerevisiae*.

## RESULTS

### Identification of Dpb3 and Dpb4 subunits of DNA polymerase epsilon in *C. albicans*

**Fig 1:**
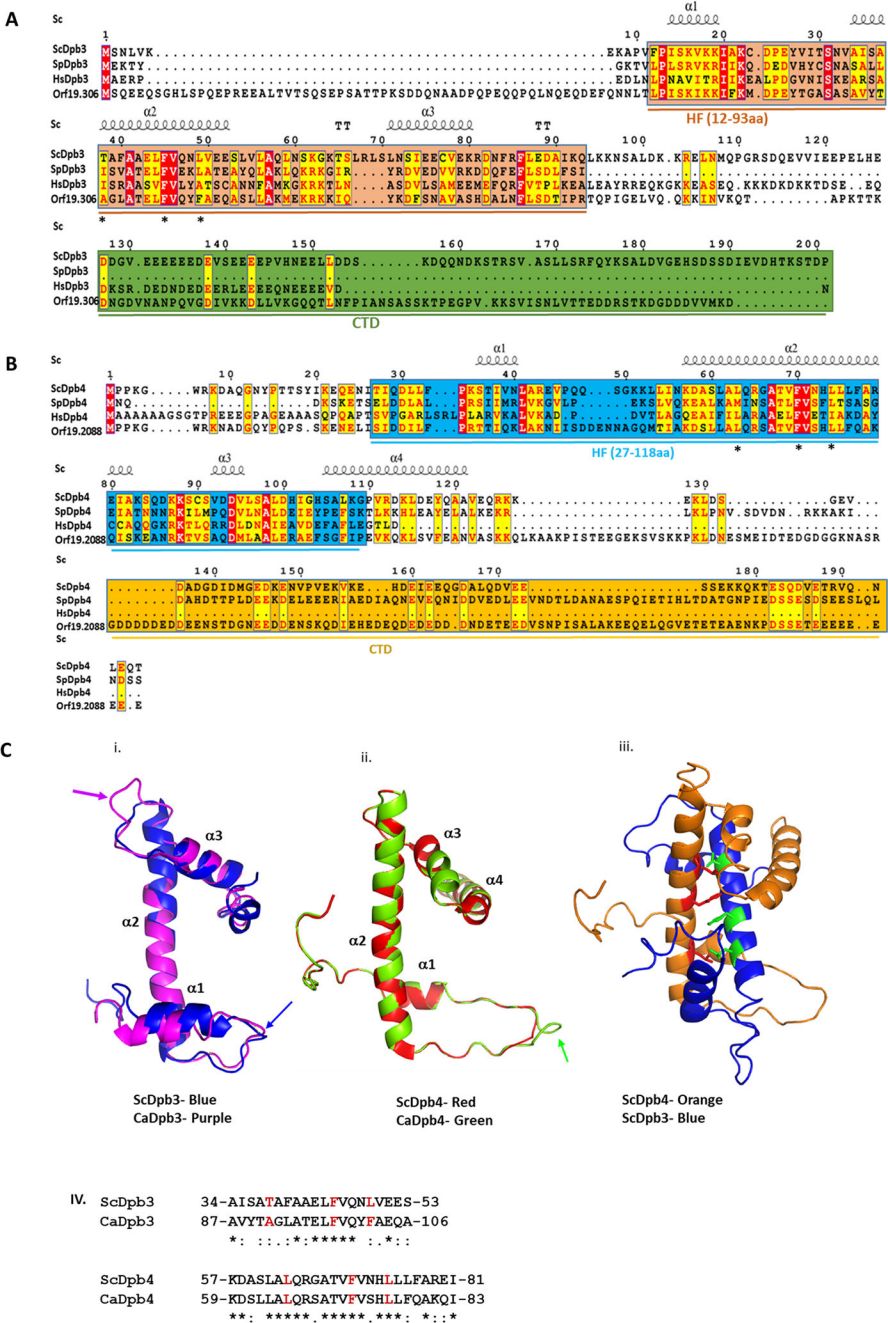
*In silico* analyses and homology modeling predicting the conserved structures of Dpb3 and Dpb4 of *C. albicans*. Multiple sequence alignment of Dpb3 (**A**) and Dpb4 (**B**) proteins from *S. cerevisiae, S. pombe, Homo sapiens,* and *C. albicans* were performed using the T-Coffee tool server. Alpha helical structures are depicted as α1, α2, α3, and α4. The histone fold (HF) and C-terminal domains were shaded with distinct colors. * depicts potential conserved interacting residues in Dpb3 and Dpb4. Homology modeling of CaDpb3 and CaDpb4 proteins was carried out using SWISS-MODEL (**C**). Superimposed images of ScDpb3 and CaDpb3 highlighted with blue and purple colors, respectively, are shown (i). The superimposed structure of ScDpb4 and CaDpb4 highlighted with red and green colors, respectively, are shown (ii). A three-dimensional image of ScDpb3-ScDpb4 showing the interacting surface and key residues involved in dimerization is provided (iii). Identification of conserved regions of CaDpb3 and CaDpb4 are likely to be involved in interaction. Red-colored amino acids are involved in Dpb3 and Dpb4 interaction in *S. cerevisiae* (iv).

The *Candida* genome database (CGD) suggests *C. albicans* harboring all the four subunits Pol2 (orf19.2365), Dpb2 (orf19.7564), Dpb3 (orf19.3063), and Dpb4 (orf19.2088) of Polε holoenzyme; however, they remain unverified so far. CGD suggests that *POL2* and *DPB2* genes are haploinsufficient in diploid *C. albicans,* and our efforts to generate heterozygous deletion strains involving these two essential genes were also unsuccessful. Since Dpb3 and Dpb4 subunits are dispensable for cell survival in *S. cerevisiae* and their role in genome stability is not fully understood*,* to begin with, we intended to characterize these subunits of *C. albicans*. Amino acid alignment revealed that the putative Dpb3 of *C. albicans* shares merely 13%–17% identity and about 20%–38% similarity with that in *S. cerevisiae*, *Schizosaccharomyces pombe*, and *Homo sapiens* (Fig. 1A and B; Table S1). Interestingly, CaDpb3 protein is composed of 237 amino acids (26 kDa), which is larger in size than ScDpb3 (201 aa, 22.6 kDa), SpDpb3 (87 aa, 10 kDa), and HsDpb3 (147 aa, 16.8 kDa). The secondary structure prediction suggests majorly the presence of three alpha helices comprising the histone-like fold (HF) in these proteins. CaDpb3 seems to possess an extra unstructured region toward the N-terminal region (7-SGH…..DEF-58), whereas both ScDpb3 and CaDpb3 possessed extended C-terminal tails. The putative CaDpb4 shares maximum homology with ScDpb4 and SpDpb4 (~ 27%–32% identity and 44%–48% similarity), but a distant similarity with HsDpb4 (10% identity and 17% similarity). Like CaDpb3, CaDpb4 (261 aa, 29 kDa) is also comparatively larger in size than ScDpb4 (196 aa, 22 kDa), SpDpb4 (210 aa, 23.5 kDa), and HsDpb4 (117 aa, 12 kDa). In contrast to Dpb3, the histone-like fold of Dpb4 subunits is comprised of four alpha helices (α1–α4). Except in humans, other Dpb4 proteins possess extended C-terminal tails. Since these accessory subunits of Polε harbor histone-like folds, we also aligned them with histone proteins of *C. albicans* (Fig. S1). CaDpb3 exhibited homology with CaH2A (23.8% similarity and 11.3% identity) and CaDpb4 showed amino acid similarly with CaH2B protein (20.6% similarity and 10.7% identity). Since we noticed additional extended regions in CaDpb3 and CaDpb4 subunits, their possible effect on the structure was verified by structural modeling using respective *S. cerevisiae* structures as templates (Fig. 1C). Both Dpb3 and Dpb4 are dumbbell-shaped comprising of helix-loop-helix-loop-helix structures. The predicted structure of CaDpb3 exhibited extraordinary structural overlapping with ScDpb3 except that the connecting loops between α1 to α2 and α2 to α3 are slightly longer in CaDpb3 (Fig. 1Ci). Similarly, the CaDpb4 model structure is also remarkably similar to ScDpb4. The loop between α1 and α2 in CaDpb4 is also longer than that in ScDpb4 (Fig. 1Cii). To find out the key residues involved in Dpb3/Dpb4 dimerization, the dimer structure of ScDpb3/ScDpb4 along with amino acid alignment was analyzed and found a conservation of A91, F98, and F102 in CaDpb3 (T38, F45, and L49 in ScDpb3), and L65, F72, and L76 in CaDpb4 (L63, F70, and L74 in ScDpb4) residues that might play a critical role in CaDpb3 and CaDpb4 interaction and function (Fig. 1Ciii and iv).

### Dpb3 and Dpb4 subunits are non-essential for survival, but their absence caused severe growth defects

**Fig 2:**
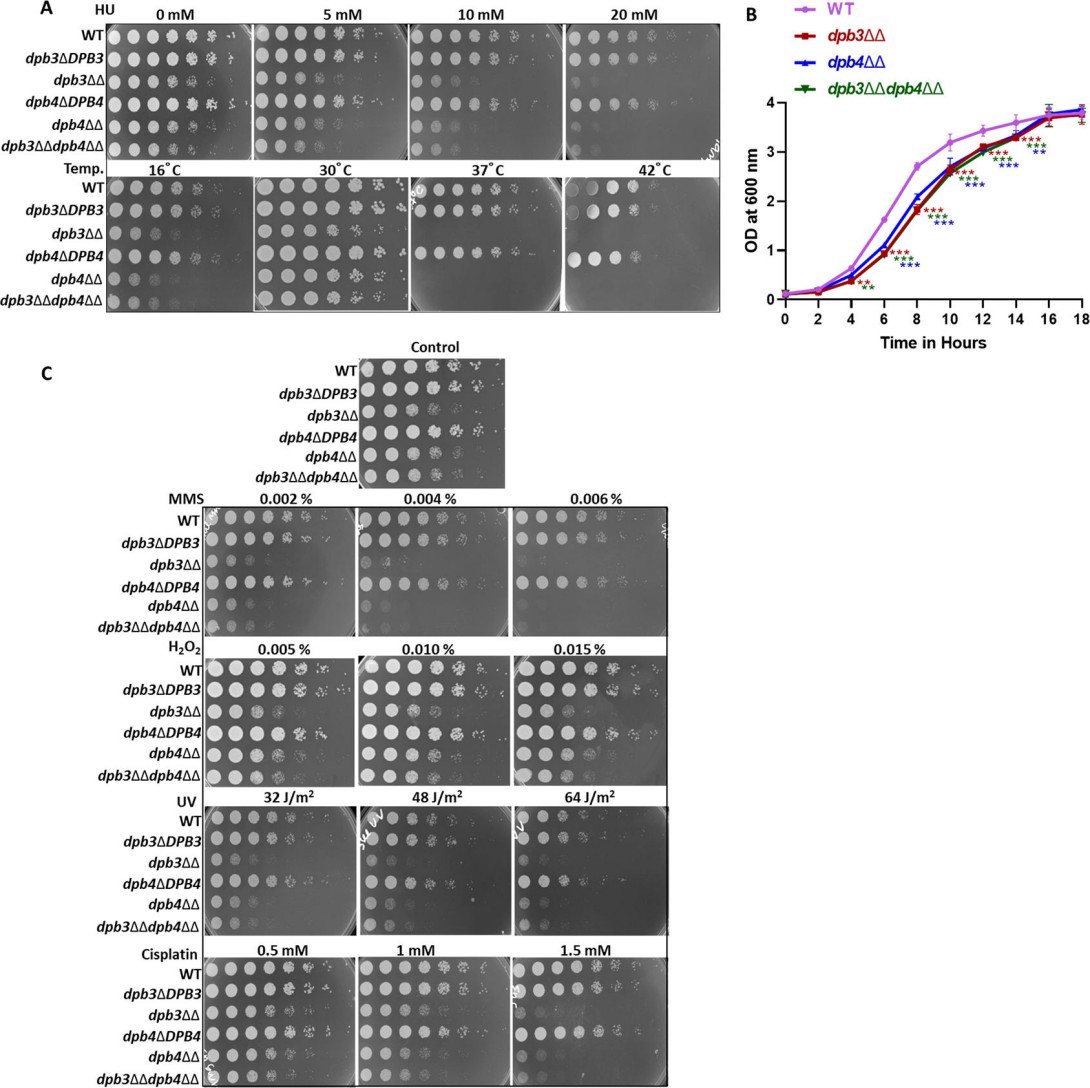
Growth curve and spot assay analyses of Polε-defective strains. The wild-type and mutant strains of *C. albicans* were serially diluted in YPD agar and spotted in the presence or absence of HU and incubated at 30°C. For temperature sensitivity, spotted plates without any genotoxic agents were incubated at 16°C, 30°C, 37°C, and 42°C. Spots were allowed to grow for 48 h and then photographed (**A**). A liquid growth curve assay was carried out for the WT (purple) and mutant strains of *C. albicans* such as *dpb3*ΔΔ (maroon), *dpb4*ΔΔ (blue), and *dpb3*ΔΔ*dpb4*ΔΔ (green) in 10 mL of YPD broth at 30°C by measuring absorbance at 600 nm at an interval of 2 h for 18 h. The experiment was carried out twice in triplicates. Asterisks indicate (***P < 0.001 and **P < 0.01) the statistically significant differences compared with the results of WT and mutant strains using two-way ANOVA with Dunnett’s multiple comparison test (**B**). Spot sensitivity test was also carried out for various strains of *C. albicans* in the presence of mentioned concentrations of MMS, H_2_O_2_, and Cisplatin. For UV treatment, completely dried spotted plates were subjected to different time exposures. Plates were allowed to grow for 48 h at 30°C and photographed (**C**).

Both in *S. cerevisiae* and *S. pombe*, Dpb3 and Dpb4 subunits are not essential for cell survival (20, 21). To investigate the role of these accessory subunits of CaPolε in fungal biology, both heterozygous and homozygous deletion strains (*dpb3*Δ*DPB3*, *dpb3*ΔΔ, *dpb4*Δ*DPB4*, *dpb4*ΔΔ, and *dpb3*ΔΔ*dpb4*ΔΔ) of *C. albicans* were generated (Fig. S2A and B). As we could achieve homozygous deletion strains of individual and both the genes, it suggested that even in *C. albicans DPB3* and *DPB4* genes are dispensable for cell survival. However, the complete lack of Dpb3 or Dpb4 or both the subunits led to slow growth phenotype in comparison to WT and heterozygous deletion strains as observed both in the spot dilution and liquid growth curve assays (Fig. 2A and B). As the absence of any of the subunits in *C. albicans* showed similar growth phenotype, it suggested that both Dpb3 and Dpb4 subunits of Polε could play an important role as a complex during DNA replication. Furthermore, the *dpb3*/*dpb4* null strains exhibited hypersensitivity to DNA replication inhibitor hydroxyurea (HU) and low/high temperatures. At 16°C and >37°C incubation temperatures, the growth of *dpb3*ΔΔ, *dpb4*ΔΔ, and *dpb3*ΔΔ*dpb4*ΔΔ strains were severely compromised, whereas the WT and respective heterozygous deletion strains of *C. albicans* grew normally. Hydroxyurea inhibits DNA replication by depleting cellular dNTPs level; thus, the sensitivity assay clearly suggested the importance of Dpb3/Dpb4 subcomplex in DNA replication. Next, we evaluated the impact of the loss of the non-essential accessory subunits of Polε during DNA damage repair by subjecting various deletion strains to DNA damaging agents (Fig. 2C). While methyl methanesulfonate (MMS) adds alkyl adducts to nitrogenous bases, H_2_O_2_ generates oxidized adducts like 8-oxoguanine in DNA. UV and cisplatin dimerize neighboring specific nucleotides that result in bulky adducts in the genome. These adducts are either repaired by various DNA repair mechanisms or tolerated via the translesion DNA synthesis process (10, 32). Our spot analyses revealed that *dpb3*ΔΔ, *dpb4*ΔΔ, and *dpb3*ΔΔ*dpb4*ΔΔ strains of *C. albicans* were more highly and equally susceptible to these genotoxic agents than the WT and respective single allele deletion strains. To strengthen it further, *DPB3* was integrated back into one of the deletion loci in *dpb3*ΔΔ strain, and susceptibility to different stressors was determined (Fig. S2C). A single copy of *DPB3* rescued the phenotypes attributed to the deletion, and *dpb3*ΔΔ::*DPB3* grew normally as the WT strain. This also confirmed no additional unwanted chromosomal deletions in *dpb3*ΔΔ that would affect the growth and the phenotypes are specific to Dpb3’s functions. Thus, both Dpb3 and Dpb4 as an individual component of the Dpb3-Dpb4 subcomplex of Polε are critical to maintaining genome stability. Interestingly, although the Dpb3/Dpb4 sub-complex was found to regulate processivity and fidelity by stabilizing the Polε holoenzyme in *S. cerevisiae,* unlike in *C. albicans,* an *S. cerevisiae* strain lacking these subunits does not show any slow growth phenotype, suggesting a diverged role of these subunits in different fungal species, thus warranting a detail investigation.

### Carboxyl terminal domains of Dpb3 and Dpb4 proteins are not required for their essential function

**Fig 3:**
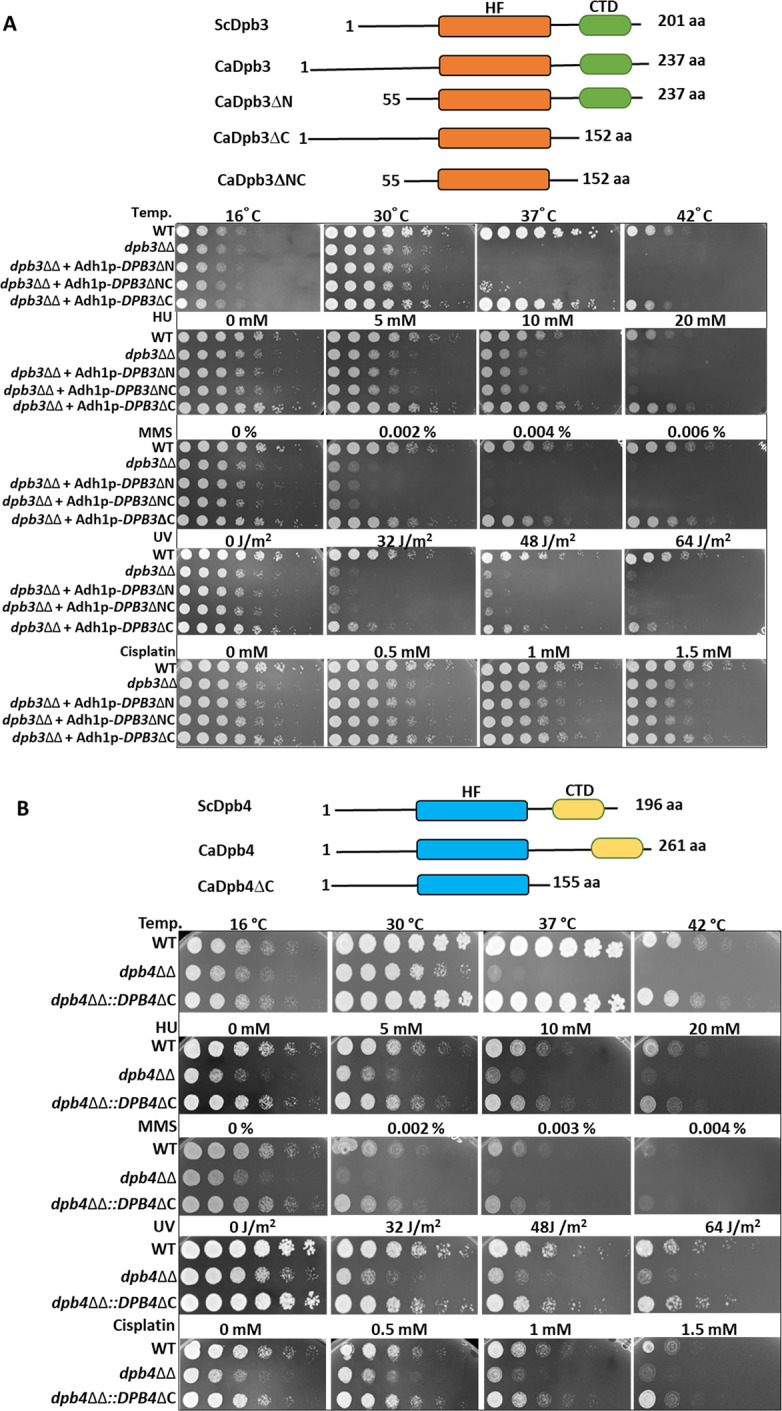
Functional analyses of extended tails of Dpb3 and Dpb4 subunits. A ray diagram showing the generation of various truncated mutants of Dpb3 protein. Spot assay of *dpb3*ΔΔ *C. albicans* strains without or with expressing CaDpb3ΔN (55–237 aa), CaDpb3ΔC (1–152 aa), and CaDpb3ΔNC (55–152 aa) proteins was carried out by spotting various dilutions of the pre-culture on YPD agar plate without or with different concentrations of HU, MMS, and cisplatin. For UV treatment, spotted plates were subjected to different times of UV-B exposure. Plates were allowed to grow for 48 h at 30°C and photographed. For temperature sensitivity, spotted plates were incubated at 16°C, 30°C, 37°C, and 42°C. Spots were allowed to grow for 48 h and then photographed (**A**). A ray diagram showing the generation of Dpb4ΔC protein. Spot assay of *dpb4*ΔΔ *C. albicans* strains without or with expressing CaDpb4ΔC (1–155 aa) protein was carried out as above and photographed (**B**).

Since the amino acid alignment of Dpb3 and Dpb4 subunits of *C. albicans* showed extended N- and C- terminal regions with minimal homology with their counterparts from other organisms, to define a role to those regions, we generated various mutant strains and analyzed by complementation analyses. For Dpb3, an amino-terminal (Dpb3ΔN 55–237 aa), a carboxyl-terminal (Dpb3ΔC 1–152 aa), and both terminus (Dpb3ΔNC 55–152 aa) were truncated and expressed under a constitutive *ADH1* promoter by integrating necessary constructs into the *RP10* locus of genome of *dpb3*ΔΔ strain of *C. albicans. RP10* locus is a neutral locus for knock-in in *C. albicans* (33). However, *DPB*4ΔC (1–155 aa) was expressed under its own promoter by integrating it into its locus in the *dpb4*ΔΔ strain of *C. albicans*. These add-back strains were exposed to extreme conditions, and survivability was determined (Fig. 3A and B). Interestingly, although the C-terminal truncated Dpb3 and Dpb4 complemented and rescued various growth defects of *dpb3*ΔΔ and *dpb4*ΔΔ strains, respectively, deletion of the first 55 amino acids of Dpb3 did not suppress such defective phenotypes despite the retention of histone-like fold. The *dpb3*ΔΔ and strains of *C. albicans* harboring Dpb3ΔN and Dpb3ΔNC exhibited similar poor cell survivability (Fig. 3A). The phenotype of Dpb3ΔNC incurred was due to the deletion of the first 55 amino acids as the Dpb3ΔC was successfully complementing the loss of Dpb3. This result suggested that the extended N-terminal tail of Dpb3 in *C. albicans* is important for certain species-specific functions of Dpb3 in stabilizing the genome.

### Heterodimerization of Dpb3 and Dpb4 subunits is critical for their role in genome stability

**Fig 4:**
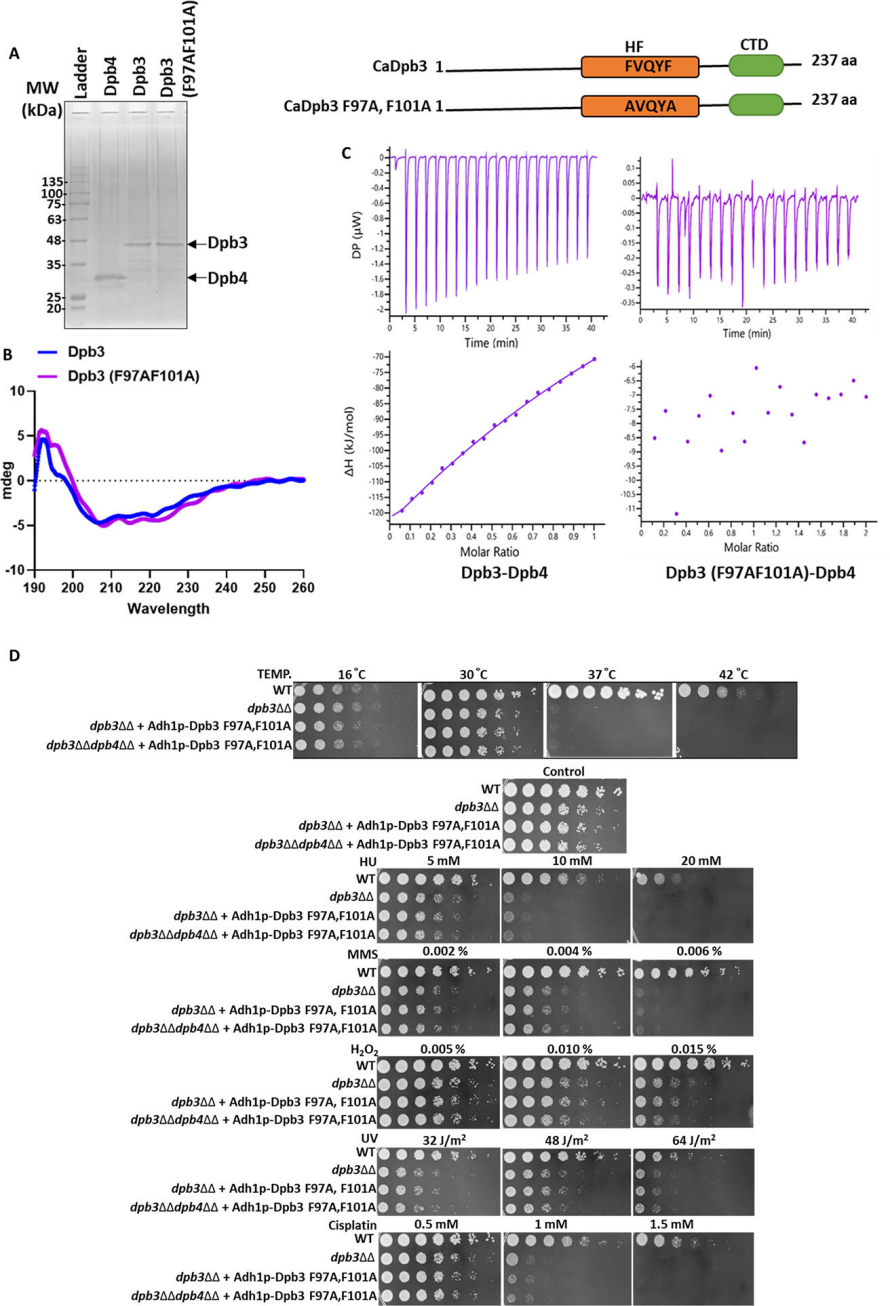
Dimerization of CaDpb3-CaDpb4 is crucial for Polε’s function in the cells. Generation and purification of His-CaDpb3, His-CaDpb3 F97A, F101A, and His-CaDpb4 proteins using bacterial expression system. Proteins were resolved on 10% SDS-PAGE (**A**). Structural integrity of CaDpb3 and CaDpb3 F97A, F101A proteins was confirmed by CD-spectroscopy. Far UV-CD spectra of wild-type (blue) and mutant Dpb3 at pH 7.5 between 190 nm and 260 nm were recorded. Data represent values determined after solvent correction and after averaging each set (*n* = 2) (**B**). Protein interaction study of Dpb3 (20 µM) with Dpb4 (100 µM) and Dpb3 F97A, F101A (20 µM) with Dpb4 (100 µM) was performed using ITC. In each figure, the upper half shows heat exchange measured during each injection of protein and the lower panel reflects enthalpy change as a function of the molar ratio of binding of two proteins (**C**). Spot assay of *dpb3*ΔΔ and *dpb3*ΔΔ*dpb4*ΔΔ *C. albicans* strains without or with expressing CaDpb3 F97A, F101A mutant was carried out by spotting various dilutions of the pre-culture on YPD agar plate without or with mentioned concentrations of HU, MMS, and cisplatin. For UV treatment, completely dried spotted plates were subjected to different time exposures. Plates were allowed to grow for 48 h at 30°C and photographed. For temperature sensitivity, spotted plates were incubated at 16°C, 30°C, 37°C, and 42°C. Spots were allowed to grow for 48 h and then photographed (**D**).

Histone-like folds are usually involved in protein-protein interaction (24). To determine whether the interaction between Dpb3 and Dpb4 is essential for the function of this subcomplex in Polε holoenzyme, first, we identified the putative key residues of Dpb3 located in the HF region that could be involved in binding to Dpb4 based on the CryoEM structure of ScPolε (17), and F97 and F101 residues of 97-FVQYF-101 motif of CaDpb3 were mutated to alanine by site-directed mutagenesis. Both wild-type and F97A, F101A mutant of Dpb3 along with wild-type Dpb4 proteins, were expressed in bacteria and purified to near homogeneity for further characterization (Fig. 4A). Although CaDpb4 migrated as per the theoretical molecular weight in SDS-PAGE, CaDpb3 migrated much slower than expected. ScDpb3 (22.6 kDa) also exhibits similar abnormal migration and resolves at a higher position than 36 kDa in SDS-PAGE (34). Circular dichroism profiles of wild-type and F97A, F101A mutant Dpb3 proteins suggested that the mutation *per se* had no effect on the overall structure, and both the proteins share an equal percentage of α helix contents (Fig. 4B). Next, the interaction of these proteins with Dpb4 was determined by isothermal calorimetry (ITC), and we found that although the wild-type Dpb3 bound strongly with Dpb4 in a 1:1 ratio with a binding constant of KD = 55 ± 20 µM, mutant Dpb3 did not show any binding as no noticeable enthalpy change was observed (Fig. 4C). After confirming the binding site, we checked the complementation ability of Dpb3 F97A, F101A mutant in the *dpb3*ΔΔ and *dpb3*ΔΔ*dpb4*ΔΔ strains. As expected Dpb3 F97A, F101A mutant did not complement the loss of any of the subunits in the *dpb3*ΔΔ*dpb4*ΔΔ strain (Fig. 4D). Since the mutant Dpb3 (F97A, F101A) failed to rescue the cell sensitivity of *dpb3*ΔΔ strain despite the presence of both the subunits of the sub-complex, it suggested that the mere presence of Dpb3 and Dpb4 subunits is not sufficient rather the interaction between the two subunits is critical for the function of the subcomplex in genome stability.

### Role of Dpb3/Dpb4 in chromosomal instability and mutagenesis

**Fig 5:**
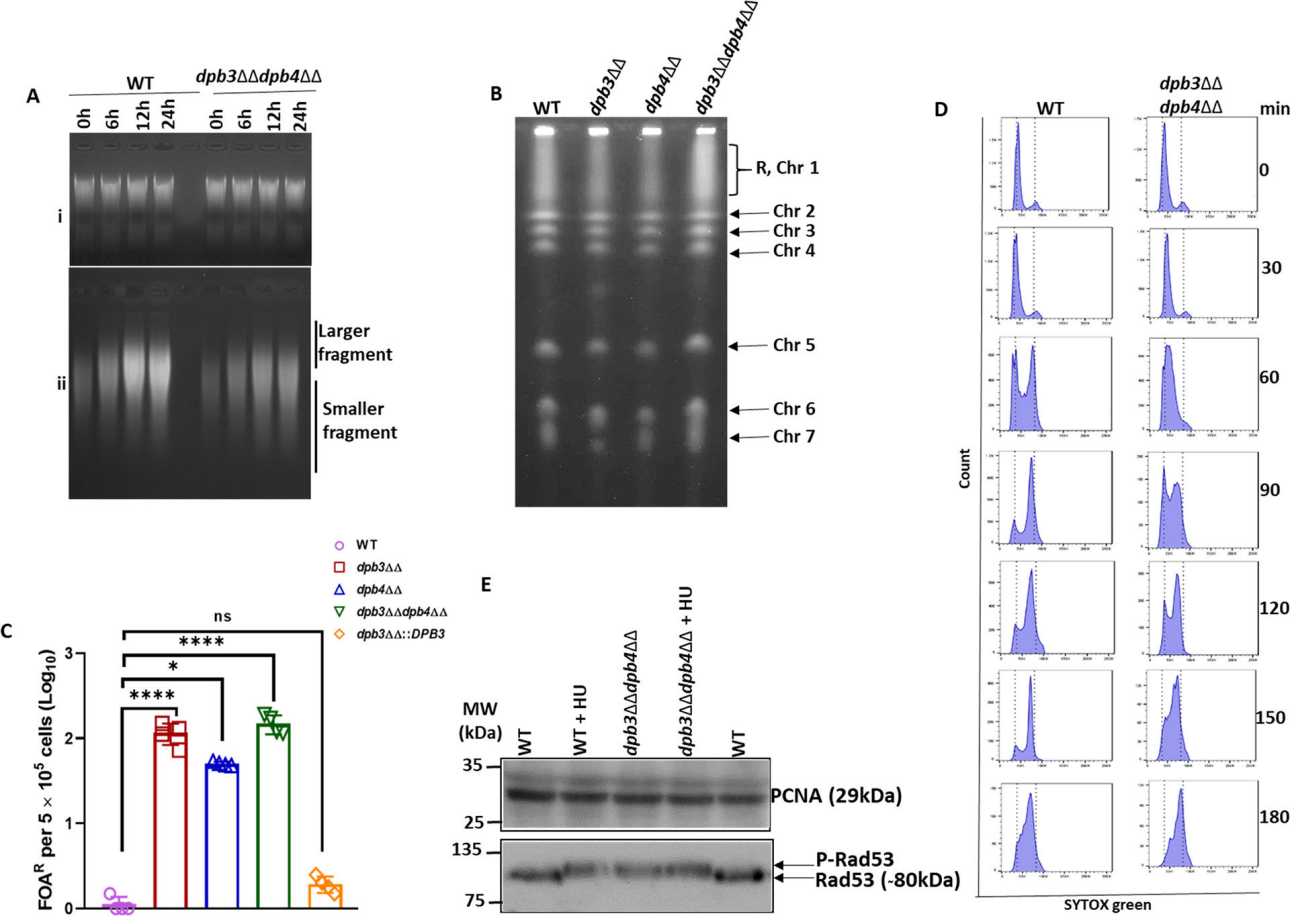
Role CaDpb3/CaDpb4 in genome stability and cell cycle progression. DNA damage recovery was carried out by exposing wild-type and *dpb3*ΔΔ*dpb4*ΔΔ cells to HU for 45 min and allowed to recover in fresh media. Total genomic DNA was isolated at various recovery time points and allowed to resolve in regular (i) and alkaline (ii) gel electrophoresis (**A**). Chromosomal karyotyping of WT and knockout strains was carried out by preparing DNA plugs and analyzing using PFGE (**B**). The rate of spontaneous mutation of various knockout strains (*dpb3*ΔΔ (maroon), *dpb4*ΔΔ (blue), *dpb3*ΔΔ*dpb4*ΔΔ (green), and WT (purple) was measured by estimating the number of FOA-resistant colonies. Mean values of four independent experiments are represented. Error bar represent SEM, and asterisks indicate (***P < 0.001, *P < 0.1, and ns-non significant) the statistically significant differences among the strains using ordinary one-way ANOVA with Tukey’s multiple comparison test (**C**). Cell cycle progression of WT and *dpb3*ΔΔ*dpb4*ΔΔ strains was monitored for 150 min by SYTOX green staining and flow cytometry after synchronizing the cells at G1 phase (**D**). Phosphorylation status of Rad53 was verified by western blotting using an anti-Rad53 antibody. The loading control was determined by anti-PCNA antibody (**E**).

Polε *per se* is a highly processive DNA polymerase and in the presence of processivity factors (RPA, RFC, and PCNA), its processive DNA synthesis increases (7). Additionally, the CryoEM structure of Polε holoenzyme suggested that Dpb3-Dpb4 dimer binds to the mooring helix of Pol2 to stabilize the complex (17). To decipher the role of Dpb3-Dpb4 in processive DNA synthesis by CaPolε, WT and *dpb3*ΔΔ*dpb4*ΔΔ strains were treated with HU for 45 min and then transferred to a fresh media to allow repairing of DNA breaks. A DNA polymerase compromised in processive DNA synthesis will fail to complete the replication of a large genome on time, leading to the accumulation of DNA breaks thereby accrual of smaller DNA fragments. Total genomic DNA was isolated at various time points of recovery and analyzed in both regular and alkaline-based agarose gel electrophoresis (Fig. 5A). Alkali-agarose gel electrophoresis revealed that an equal amount of genomic DNA degradation was observed in both of the strains prior to recovery at 0 h time point (Fig. 5Aii; Fig. S3). However, with an increase in the duration of the recovery period, more and more accumulation of larger DNA fragments was observed; consequently, a lesser amount of smaller chromosomal DNA fragments was detected in WT than in *dpb3*ΔΔ*dpb4*ΔΔ strain (Fig. S3, compare blue vs purple). The accumulation of smaller-sized fragmented DNA in *dpb3*ΔΔ*dpb4*ΔΔ *C. albicans* strain even after 24 h of recovery phase suggested a critical role of Dpb3-Dpb4 subcomplex in Polε’s function during DNA replication in the cell. The normal agarose gel electrophoresis of total genomic DNA ensured the analysis of an equal amount of DNA for each cell type and duration (Fig. 5Ai). Further to check the effect of defective Polε’s activity due to the loss of Dpb3-Dpb4 on gross chromosomal alteration, we carried out pulse field gel electrophoresis (PFGE) analyses of total genomic DNA and found that all the eight pairs of chromosomes were intact, and no apparent abnormal chromosomal mobility was detected in any of the *C. albicans* strains (WT, *dpb3*ΔΔ, *dpb4*ΔΔ, and *dpb3*ΔΔ*dpb4*ΔΔ). Thus, the loss of the accessory subunits of Polε did not induce any noticeable karyotypic alteration in these cells (Fig. 5B). Although there was no effect on the gross chromosomal instability and as the DNA synthesis was compromised due the absence of Dpb3-Dpb4 subunits, we estimated the rate of spontaneous mutagenesis in the deletion strains of *C. albicans* by using a counter-selectable *URA3* gene marker. For that, one allele of the *URA3* gene was deleted in various genetic backgrounds, and the loss of the functional copy was verified by subjecting the strains to 5FOA. Cells survive in the presence of 5FOA only when the other *URA3* allele becomes nonfunctional, and the number of 5FOA^R^ colonies suggests the rate of spontaneous mutagenesis (Fig. 5C). Consistent with our earlier reports, the mutation rate was bare minimum (~3–4 colonies per 5 × 10^5^ cells) in the wild-type and *dpb3*ΔΔ::*DPB3* complemented strains (31, 35); however, in the absence of both or any of the subunits of Dpb3-Dpb4 complex, the mutation rate was elevated by 20-fold to 100-fold, suggesting a critical role of Dpb3-Dpb4 complex in Polε’s fidelity. Somehow, we observed a higher rate of spontaneous mutagenesis rate in *dpb3*ΔΔ and *dpb3*ΔΔ*dpb4*ΔΔ strains than that in *dpb4*ΔΔ.

### Loss of Dpb3/Dpb4 subunits delays cell-cycle progression and activates Rad53 phosphorylation

In *S. cerevisiae*, the *dpb4*Δ cells showed a prolonged S phase in comparison to its isogenic wild type (36). Since the loss of Dpb3-Dpb4 subcomplex resulted in a slow growth phenotype and compromised DNA synthesis, it may also play a role in cell cycle progression. To monitor it, cells of WT and *dpb3*ΔΔ*dpb4*ΔΔ strains were synchronized at the G1 phase, and cell cycle analysis was carried out (37). Cells were harvested at different time points, and DNA was stained with SYTOX green. The cell cycle progression of the gated single-nucleated cells was monitored by flow cytometry (32). Although the WT cells completed a cycle of 2N (G1) to 4N (G2/M) in ~90 min, a significant delay was observed in *dpb3*ΔΔ*dpb4*ΔΔ cells as most of them were still in the S phase for the same duration of progression analysis. The *dpb3*ΔΔ*dpb4*ΔΔ cells took close to 120 min to complete a cycle (Fig. 5D). DNA replication checkpoint pathways prevent cells with incompletely replicated DNA from entering mitosis, and in response to replication stress, yeast cells activate *MRC1* that encodes for a signal transducer, which further activates *RAD53*, a downstream effector kinase of checkpoint. HU stimulates phosphorylation of Rad53 (38); by western analysis, we also found a slower migrating phospho-Rad53 upon treatment with HU in WT *C. albicans* than in untreated cells (Fig. 5E). However, a constitutive phospho-Rad53 was found in *dpb3*ΔΔ*dpb4*ΔΔ cells. Altogether, these results suggested that although Dpb3-Dpb4 subunits are non-essential for growth, their absence results in replication stress to activate the replication checkpoint to minimize genome instability.

### Comparative genomics of *dpb3*ΔΔ*dpb4*ΔΔ and its isogenic parental strain of *C. albicans*

**Fig 6:**
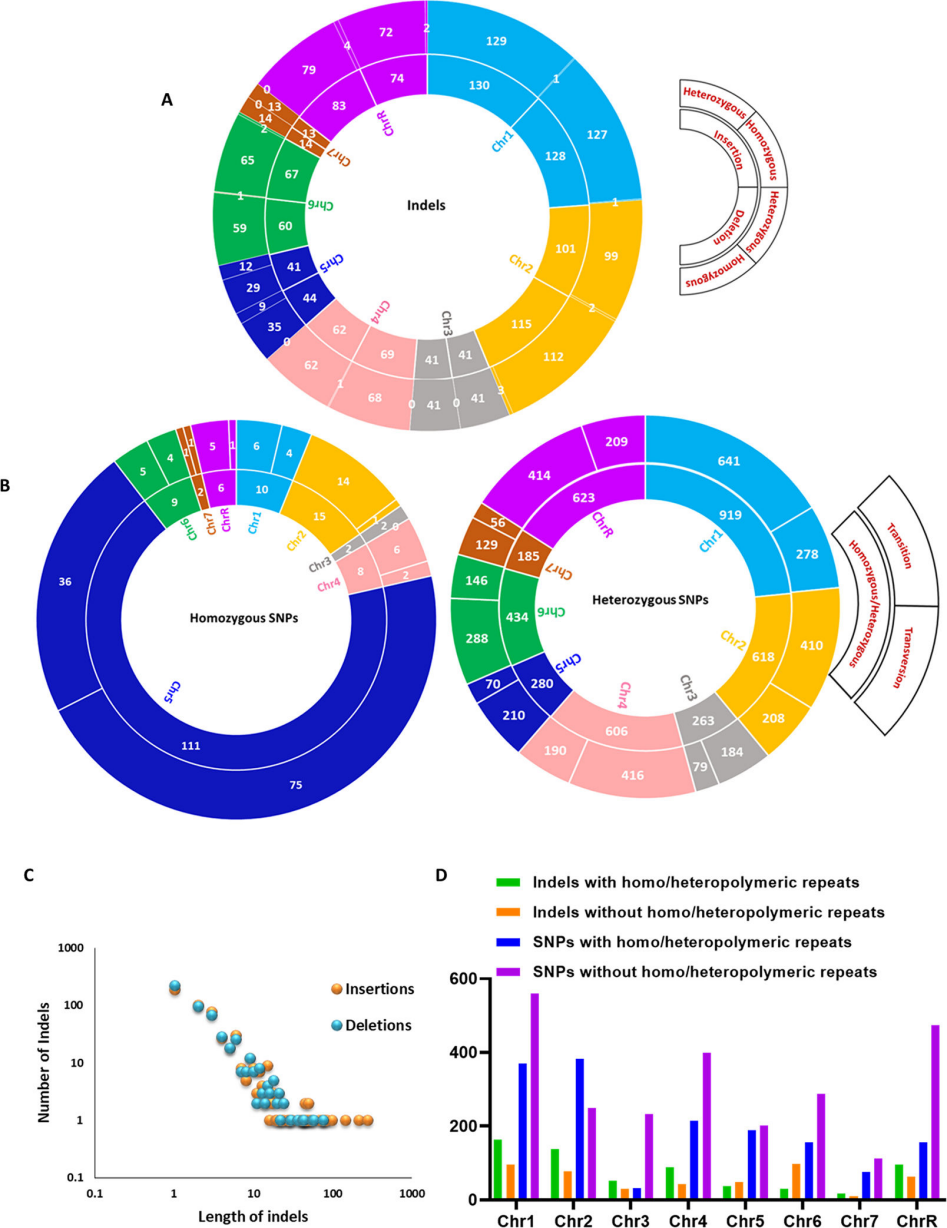
Whole genome sequencing of *dpb3*ΔΔ*dpb4*ΔΔ strain of *C. albicans*. A double-ring pie-chart showing accumulation of indels (insertion and deletion) in each chromosome of *dpb3*ΔΔ*dpb4*ΔΔ strain where the inner ring depicts insertions (left) and deletions (right), and the outer ring suggests their presence in one or both the alleles. For example, Chr 1 has 130 insertions, of which 129 are heterozygous and one variation is homozygous. Similarly, Chr 1 has 128 deletions, of which 127 are heterozygous and one variation is homozygous (**A**). A double-ring pie-chart showing accumulation of homozygous and heterozygous SNPs in each chromosome of *dpb3*ΔΔ*dpb4*ΔΔ strain where the inner ring depicts total homozygous or heterozygous SNPs, and the outer ring suggests the number of transition (left) and transversion (right) variations. For example, Chr 1 has 10 homozygous SNPs, of which six are transition and four are transversion mutations (**B**). The frequency of distribution of length of deletions and insertions is specific to *dpb3*ΔΔ*dpb4*ΔΔ genome (**C**). Accumulation of indels and SNPs with or without homo/heteropolymeric repeat regions in *dpb3*ΔΔ*dpb4*ΔΔ genome (**D**).

To get further insights into the role of the Dpb3-Dpb4 subcomplex in genome stability, whole-genome sequencing of the *dpb3*ΔΔ*dpb4*ΔΔ as well as the isogenic wild-type strain of *C. albicans* were performed and compared. After quality controls of the raw reads, a total of 259 and 98 contigs corresponding to 14,528,623 and 14,456,421 bp of total reads with N50 of 2,140,869 and 2,161,857 bp were obtained for wild-type and *dpb3*ΔΔ*dpb4*ΔΔ genome sequence, respectively*,* and the total genome sizes are very much comparable with the reference genome size (ASM18296v3). The genome sequencing confirmed the deletion of *DPB3* and *DPB4* genes in Chr 1 and Chr 2, respectively, and detailed chromosome-wise genetic changes specifically accumulated in the *dpb3*ΔΔ*dpb4*ΔΔ strain after subtracting the common variations were illustrated (Fig. 6A and B; Tables S2 and S3). In addition to the two known gene deletions, 1,083 indels (542 insertions + 541 deletions) and 4,091 SNPs were identified in the *dpb3*ΔΔ*dpb4*ΔΔ genome. These deletions and insertions vary from one to ~279 bp, and large-sized insertions were relatively more accumulated than large-sized deletions (Fig. 6C). Of which only 38 indels and 163 SNPs were found in both the alleles. Except for 3, the rest of the homozygous indels were upstream genetic variants and less likely to affect gene functions. The three indels caused frameshift mutations in so far uncharacterized orfs (Table S4). Chromosomal features are known to govern mutational patterns in eukaryotic organisms. Repetitive regions, sub-telomeric regions, and similar gene families are the hotspots where the mutation spectrum alters frequently. Based on the size of the chromosomes, a large number of indels and SNPs were accumulated. Although Chr 3 is relatively a large chromosome [1.8 MB, whereas the sizes range from 3.1 (Chr 1) to 0.9 MB (Chr 7)], it accumulated a lesser number of such variations, probably as it does not possess any major repeat sequence (MRS) regions that are known to cause replication slippage. Although Chr 3 was the most stable in the genome, Chr 6 (1 MB), which possesses 5 MRS regions, was prone to accumulate more variations in the *dpb3*ΔΔ*dpb4*ΔΔ background. Further analyses revealed that of 1,083 indels, about 60% of indels (622) were located in the homo- and hetero-polymeric repeat regions of the chromosomes (Fig. 6D; Table S2 and Fig.S4). Again, ~50% of SNPs were identified in the repeat regions (Table S3). This suggests that more than the fidelity, it is the processivity of Polε that gets affected in the absence of the Dpb3-Dpb4 subcomplex and contributes to high rates of indel accumulation in the repeat sequences. These genetic variations in *dpb3*ΔΔ*dpb4*ΔΔ could influence the phenotype and pathogenesis of *C. albicans*.

### *C. albicans* strains defective in the accessory subunits of Polε exhibit constitutive filamentation and altered virulence-associated gene expression

**Fig 7:**
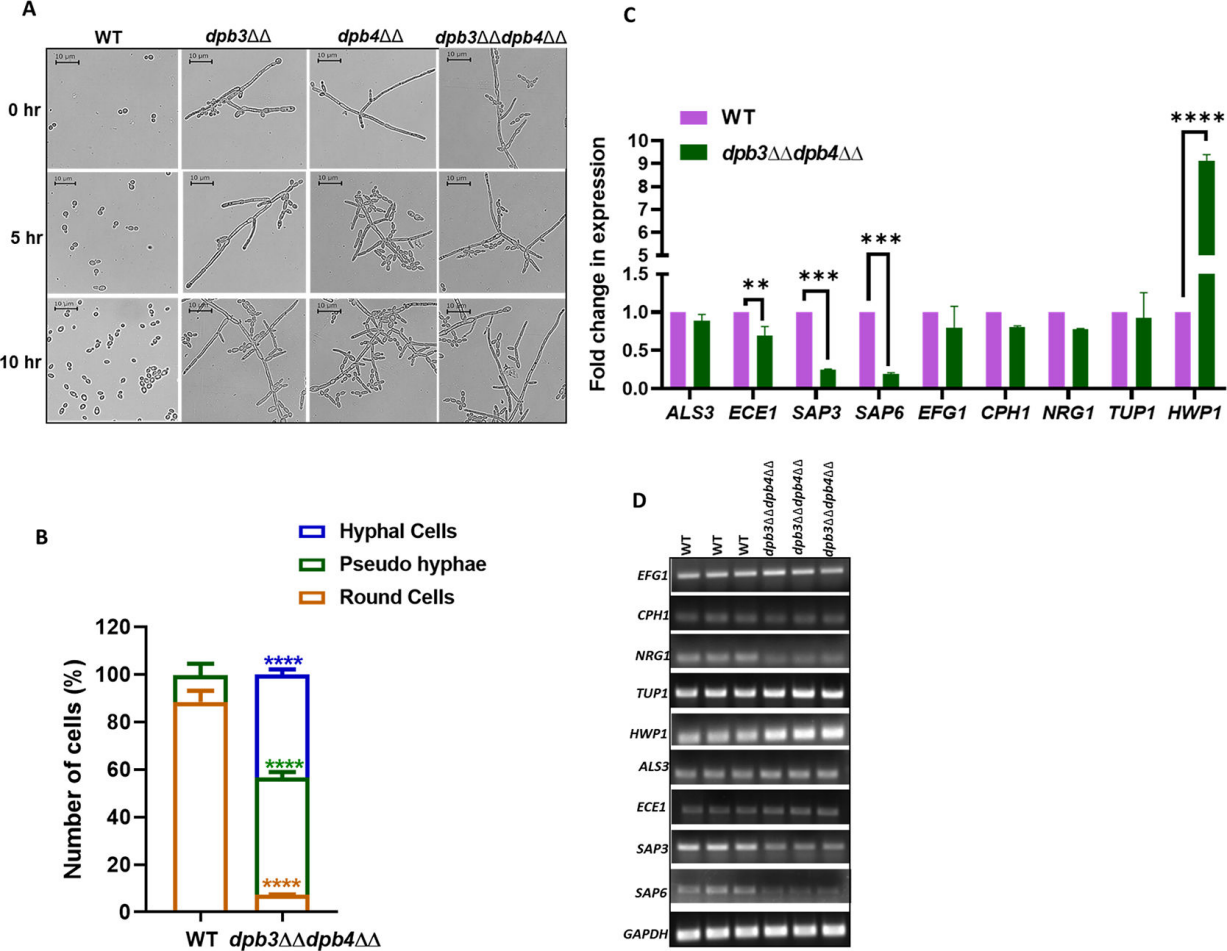
Morphogenesis and virulence determinants of *dpb3*ΔΔ*dpb4*ΔΔ strain of *C. albicans*. Various knockout strains and WT were grown in YPD broth at 30°C for 10 h, and the morphology of cells was observed at mentioned time points under a light microscope using 40× magnification (**A**). Percentage of various forms of cells was quantified after 1 h of inoculation at 30°C. Multiple focuses were taken into account to count the number of different morphology of cells. Mean values of three independent sets were represented. Error bars represents SEM, and asterisks indicate (***P < 0.001) the statistically significant differences among the strains using ordinary one-way ANOVA with Tukey’s multiple comparison test. Hyphal cells (blue), pseudo hyphae (green), and round cells (orange) were counted (**B**). Gene expression study was carried out by qRT-PCR. The expression of genes associated with virulence and hyphal morphogenesis was measured using SYBR green. GAPDH was used as control. ΔΔct values were calculated and compared for the WT and *dpb3*ΔΔ*dpb4*ΔΔ strains. Calculated fold change was plotted using GraphPad Prism 8 software. Mean values from three independent experiments are shown, and error bars represent the SEM. Statistical analysis was performed using two-way ANOVA, and asterisks indicate statistically significant differences compared with the results for the WT using Sidak’s multiple comparison test (∗*P* < 0.05; ∗*P* < 0.01; ∗< 0.001; and ∗*P* < 0.0001) (**C**). A semi-quantitative reverse transcription PCR of virulence gene expression in WT and *dpb3*ΔΔ*dpb4*ΔΔ strains was determined and observed in agarose gel electrophoresis (**D**).

Although the wild-type *C. albicans* cells are majorly round-shaped under normal physiological conditions, in the presence of inducers like serum, high temperature, etc, they undergo morphological transition to form pseudohyphae and hyphae. Genomic stress due to inadequate cellular dNTPs and accumulation of DNA lesions, defects in DNA replication, delay in cell cycle progression, and activation of checkpoints also regulate morphological transitions in *C. albicans* (39). As these defects were apparent in *dpb3*ΔΔ, *dpb4*ΔΔ, and *dpb3*ΔΔ*dpb4*ΔΔ strains, their morphology was examined (Fig. 7A and B). To our surprise, these cells did not require any chemical inducer and instantly developed into hyphal-like structures, whereas the WT *C. albicans* cells showed oval-shaped structures. Thus, we concluded that Polε-defective strains due to the loss of any of the accessory non-essential subunits are constitutively filamentous. The morphological transition of *C. albicans* is due to, or leads to, altered expression of several genes associated with fungal virulence-like transcription factors involved in filamentation, agglutinin-like sequence (Als) required for cell-surface adhesion, and secreted aspartyl proteases (Sap) and Ece1 for membrane penetration, etc (40–42). Although *EFG1* and *CPH1* are the transcriptional activators, *NRG1* and *TUP1* are the transcriptional repressors of filamentation (43). *HWP1* is a downstream hyphal-specific virulence gene. *ECE1* encodes a cytolytic peptide toxin that causes damage to epithelial membranes, triggers a danger response-signaling pathway, and activates epithelial cell immunity (44). The expression analyses as examined by both real-time and semi-quantitative reverse transcription PCRs implied that although *HWP1* was upregulated by ~9-fold, expression of genes like *ECE1, SAP3,* and *SAP6* was drastically reduced in *dpb3*ΔΔ*dpb4*ΔΔ strain. The mRNA levels of other genes were unaltered (Fig. 7C and D). To verify any mutation that caused a differential expression of *HWP1, ECE1, SAP3,* and *SAP6* genes, careful validation of the whole genome sequence of *dpb3*ΔΔ*dpb4*ΔΔ strain did not reveal any such genetic variations in these genes. Thus, the differential transcriptional alternation is not due to acquired mutations but rather could be a downstream effect of replication stress response in Polε-defective strain.

### Polε-defective strains of *C. albicans* are avirulent in mice model of systemic candidiasis

**Fig 8:**
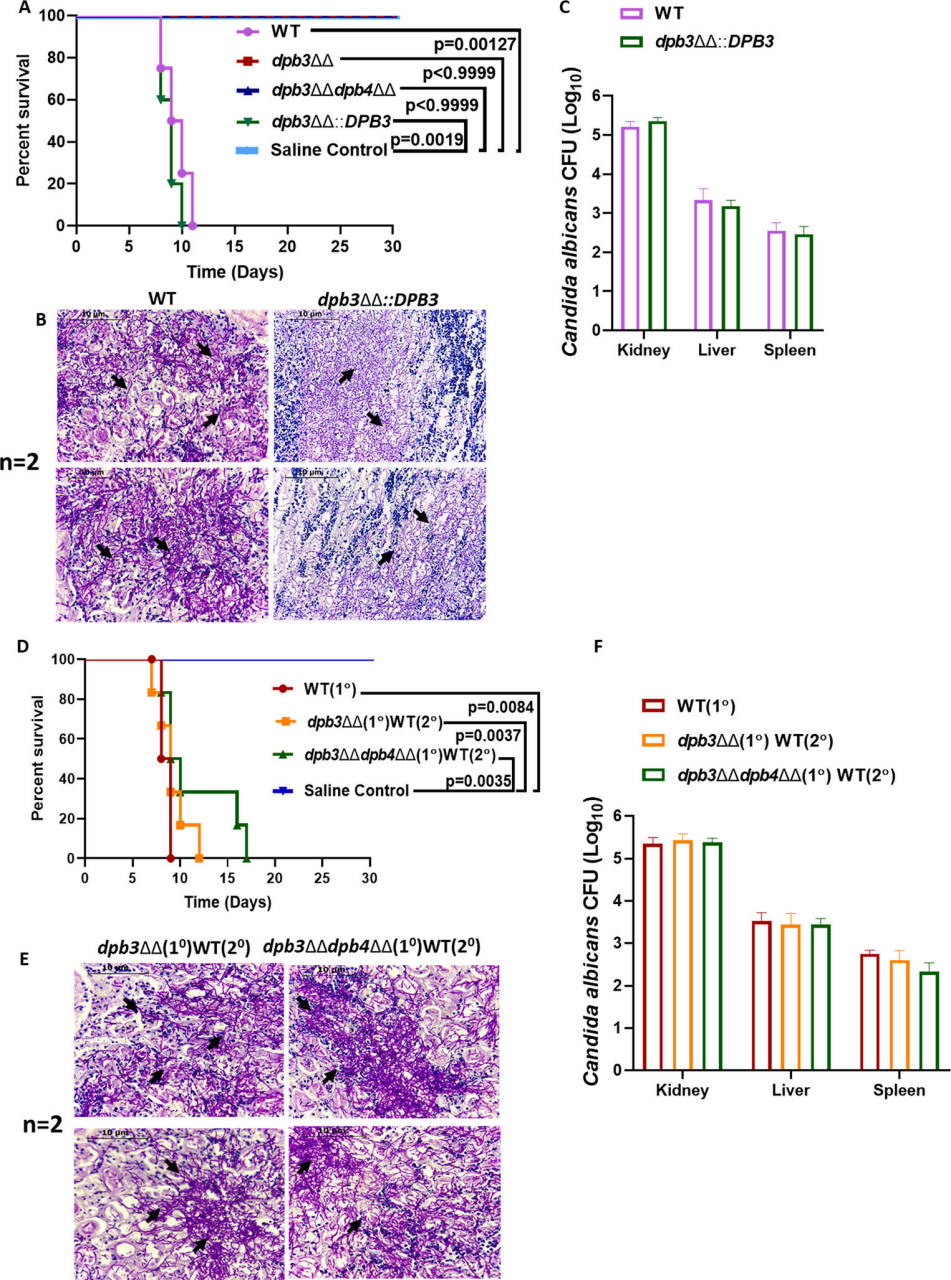
Systemic candidiasis development in mice model. Survival curve of BALB/c mice (*n* = 6) after inoculating 5 × 10^5^ CFUs of WT, *dpb3*ΔΔ, *dpb3*ΔΔ::*DPB3*, and *dpb3*ΔΔ*dpb4*ΔΔ strains of *C. albicans* intravenously. About 100 µL of saline was intravenously injected into the control group, and survivability was monitored for up to 30 days. Graph was plotted using GraphPad Prism 8 software. Data are representative of two different sets of experiments and are analyzed using the log-rank (Mantel-Cox) test. The actual *P* values are listed in the graph as per the corresponding comparison groups (**A**). The PAS staining of kidney sections (n=2) shows the fungal hyphae as indicated by arrows. Unstained portion of the kidney images may look similar (**B**). Fungal burden in kidney, spleen, and liver was estimated by CFU count on YPD + chloramphenicol plates. Mean values from three independent experiments were shown, and error bars represent the SEM (**C**). BALB/c mice (*n* = 6) were immunized with 5 × 10^5^ CFUs of *dpb3*ΔΔ or *dpb3*ΔΔ*dpb4*ΔΔ strains (1°). After 30 days, mice were re-challenged with WT (2°). In control experiments, six mice each were challenged with WT and saline, and survival was monitored. Graph was plotted using Graphpad Prism 8 software. 1° is primary, and 2° denotes re-challenge. Data are the representative of two different sets of experiments and are analyzed using the log-rank (Mantel-Cox) test. The actual *P* values are listed in the graph as per the corresponding comparison groups (**D**). The PAS-stained kidney sections (n=2) showed the fungal hyphae as indicated by arrows (**E**). Fungal burden in the kidney, spleen, and liver was estimated by CFU count on YPD + chloramphenicol plates. Mean values from three independent experiments were shown, and error bars represent the SEM (**F**).

Downregulation of some of the virulence candidate genes in filamentous *dpb3*ΔΔ*dpb4*ΔΔ strain of *C. albicans* compelled us to determine the disease-causing ability of Polε-defective strains in a mouse model of hematogenously disseminated candidiasis. In this model, upon systemic administration of *C. albicans* cells, animals die due to severe sepsis with the highest fungal load in the kidneys; the phenotype mimics the severe human fungal infection cases. The BALB/c female mice (*n* = 6) were injected with a fungal dose of 5 × 10^5^ CFU of *dpb3*ΔΔ, *dpb3*ΔΔ::*DPB3*, *dpb3*ΔΔ*dpb4*ΔΔ, and wild-type *C. albicans* cells per mouse via the lateral tail vein, and their survivability was observed for 30 days (Fig. 8A). Similarly, a mice group (*n* = 6) was injected with the same volume of saline as control. Mice that suffered due to severe candidiasis, based on humane endpoints, were euthanized. The mice challenged with WT and nearly isogenic *dpb3*ΔΔ::*DPB3* strain succumbed to infection within 11 days of inoculation, whereas 100% of mice challenged with *dpb3*ΔΔ and *dpb3*ΔΔ*dpb4*ΔΔ strains survived as good as the saline group and did not develop any noticeable symptoms. The CFU analysis of the kidney, liver, and spleen and histopathology of the PAS-stained kidney of mice of the WT and *dpb3*ΔΔ::*DPB3* challenged group confirmed the cause of morbidity due to the presence of high fungal burden in vital organs (Fig. 8B and C). As usual, the highest fungal load was detected in the kidney tissue (~2 to 3 × 10^5^ cells), followed by that in the liver and spleen (~2 to 6 × 10^2^ cells). The surviving mice were allowed to have their natural death. This result demonstrated that the *C. albicans* strains defective in the non-essential accessory subunit(s) of Polε are avirulent, and upon immunization, it may protect against the secondary challenge of fungal infections.

### Immunization with Polε-defective strains of *C. albicans* did not protect the lethal re-challenge

The result that the deletion of *DPB3-DPB4* genes of *C. albicans* attenuated the virulence to become completely non-pathogenic, hence, we argued that *dpb3*ΔΔ or *dpb3*ΔΔ*dpb4*ΔΔ strains could generate robust protective immune responses as a whole cell vaccine candidate in animals to protect the future lethal re-challenge of virulent *C. albicans*. To validate it, groups of BALB/c mice (*n* = 6) were first immunized intravenously with 5 × 10^5^ cells of these avirulent strains or 100 µL of saline per mouse as sham immunized control, and post 30 days of primary challenge (1°), they were re-challenged (2°) with wild-type *C. albicans* (5 × 10^5^ cells per mouse), and survivability was monitored (Fig. 8D). Interestingly, although all the primary challenged mice survived again confirmed avirulent attributes of the Polε defective strains, the immunized mice did not show any protection to WT re-infection, and mice succumbed within 12 days of re-challenge. The CFU analysis of the kidney, liver, and spleen and PAS-stained kidney of dead mice confirmed the cause of death due to fungal load in the vital organs (Fig. 8E and F). We repeated the experiment with a higher dose of immunization with *dpb3*ΔΔ*dpb4*ΔΔ cells (6 × 10^6^ cells), and after 30 days, they were re-challenged with 5 × 10^5^ cells of WT *C. albicans*. Despite a higher load of primary inoculation, mice did not survive to WT re-challenge (Fig. S5). Since the Polε-defective strains are temperature-sensitive at 37°C, most likely these avirulent strains did not replicate enough in the host to elicit protective immunity; therefore, they failed to protect against virulent *Candida,* thus, less likely to be explored for live whole-cell vaccine development. Nevertheless, Dpb3-Dpb4 subunits of Polε play a critical role in DNA replication, genome stability, virulence, and pathogenesis of *C. albicans,* and the interacting surface between Dpb3 and Dpb4 can be targeted for antifungal drugs. More importantly, temperature sensitivity could be one of the critical parameters to be considered to test the pathogenicity and immunogenicity of genetically modified *C. albicans* strains.

## DISCUSSION

DNA Polε, being a major eukaryotic DNA polymerase, plays an important role in stabilizing a cell’s genome. Mutational analyses of all four subunits of Polε have been carried out in yeasts, and various disease-associated mutations in Pol2 were also mapped to determine the importance of each subunit in mutagenesis, carcinogenesis, and genome stability. For example, the deletions of the N-terminal region or point mutations in the polymerase active site of the *POL2* gene in *S. cerevisiae* and *S. pombe* that lead to an inactivation Polε’s catalytic activity do not cause lethality (11, 45–47). Although the *S. cerevisiae* strain lacking Polε exonuclease and polymerase activities (*pol2-16*) survives, it exhibits severe growth defects, activation of S-phase checkpoint, increased cellular level of dNTP, and enhanced mutation rates (14). Despite Polε being a leading strand DNA polymerase, *pol2* exo- mutant (*pol2-4*) shows a weaker mutator phenotype than its corresponding Polδ mutant strain (pol3 exo-), and although a strain with both *pol2*-exo- and mismatch repair defect survives, *pol3*-exo- with mismatch repair-defective strains are not viable (48). A single amino acid substitution P286R in the exonuclease domain of Pol2 has been identified as an ultramutator, and the transgenic mice with Polε P286R rapidly develop lethal cancers of diverse lineages (49). A number of temperature-sensitive *DPB2* mutant strains of *S. cerevisiae* have been analyzed, and several of the *dpb2* alleles are highly mutagenic. The mutation rates are equivalent to *pol2-4* or *msh6* mutants. They were also defective in interaction with Pol2 (50, 51). Although the fidelity of both Polε holoenzyme with and without Dpb3-Dpb4 subcomplex (Pol2-Dpb2) is comparable, loss of *DPB3* and *DPB4* elevates spontaneous frameshift and base substitution rates in *S. cerevisiae* as good as in *pol2-4* strain (20). All these reports clearly advocated equally important roles for both essential and non-essential subunits of Polε in genome stability.

*Candida* genome database (CGD) suggests the presence of all four genes of Polε (*POL2*: CR_07,000C_A; *DPB2*: CR_09,900C_A; *DPB3:* C1_03,640C_A; and *DPB4:* C2_00,430C_A), thus, *C. albicans* most likely possesses a tetrameric holoenzyme. Interestingly, although in *S. cerevisiae,* all four genes are distributed in four different chromosomes, *POL2* and *DPB2* co-exist in Chr R, the second largest chromosome of *C. albicans*. Since Polε is yet to be characterized in any *Candida* species, to begin with, we generated a series of deletion/mutant strains pertaining to *DPB3* and *DPB4* genes of *C. albicans*. As we could readily obtain heterozygous and homozygous deletants of *DPB3* and *DPB4* and complete loss of both of these genes in *C. albicans,* it suggested that similar to *S. cerevisiae*, these genes are dispensable for growth, and cells can survive with *POL2* and *DPB2* genes. According to CGD, *C. albicans* show haploinsufficiency for *POL2* and *DPB2* genes, suggesting their indispensable nature. Interestingly, a haploid *S. cerevisiae* strain (*a* and *α*) survives and shows an almost similar phenotype as the diploid strain. It implements that Polε might be involved in additional essential functions in *C. albicans*. Unlike in *S. cerevisiae* (20, 36), *dpb3* and *dpb4* null strains of *C. albicans* exhibited slow growth and temperature-sensitive phenotype. Additionally, *dpb3*ΔΔ, *dpb4*ΔΔ, and *dpb3*ΔΔ*dpb4*ΔΔ strains were equally sensitive to HU and DNA-damaging agents. It suggested that Dpb3 and Dpb4 subunits cannot complement each other, and the presence of both subunits only forms a functional complex. Therefore, a mutant of Dpb3 (F97A, F101A) that is defective in interaction with Dpb4 failed to rescue the phenotypes exhibited by *dpb3*ΔΔ cells. Alkali agarose gel-based DNA synthesis recovery assay revealed that DNA synthesis is compromised in the *dpb3*ΔΔ*dpb4*ΔΔ strain. Altogether, this information suggested that although the Dpb3-Dpb4 complex is non-essential for survival, it plays a critical role in DNA replication. The FOA-mutagenesis assay and whole-genome sequencing revealed an enhanced rate of spontaneous mutation in *dpb3/dpb4* null strains. Although PFGE electrophoresis did not reveal any gross chromosomal disarrangement, accumulation of localized indels was quite evident in the genome. Homo- and hetero-repeat regions of the genome accumulated more insertions, suggesting replication slippage due to the absence of Dpb3-Dpb4 subcomplex compromising Polε’s fidelity. In addition, a delay in cell cycle progression and activation of Rad53 kinase were observed in the *dpb3/dpb4* null strain. Thus, the Dpb3-Dpb4 subcomplex contributes to genome stability. Upon genotoxic stress such as due to treatment with HU or MMS, *C. albicans* cells exhibit delayed cell cycle progression, phosphorylation of the effector kinase Rad53, and hyphal transition, and these phenotypes are interlinked (38, 52, 53). Accordingly, the deletion of *rad53* does not cause filamentation in *C. albicans* upon genotoxic stress exposure (39). Since the *dpb3/dpb4* null strains are defective in DNA replication, we observed similar phenotypes concurrently even without treatment with genotoxic agents. This study again confirmed the DNA integrity and cell cycle networks constituting a parallel branch of regulators involved in the morphological transition of *C. albicans*. Similar to the Dpb3-Dpb4 subcomplex of Polε, Dpb4 also dimerizes with Dls1 to form a complete ISW2 chromatin-remodeling complex to play a role in DSB (23, 24). To our surprise, the Dls1 homolog is intrinsically absent in *C. albicans*; thus, the composition and function of the ISW2 chromatin-remodeling complex in *C. albicans* might differ. The genomic stress also led to the downregulation of some of the virulence determinants like Ece1, Sap3, and Sap6 in *dpb3/dpb4* null strains. Accordingly, the *dpb3/dpb4* defective strains were non-pathogenic and failed to induce systemic candidiasis in the pre-clinical model. Since most of the occurrence of indels and SNPs events were limited to the intergenic regions of the genome and *DPB3* integration in *dpb3*ΔΔ strain was as virulent as the WT cells, the acquired genomic alterations due to the loss of Dpb3-Dpb4 subunits have minimal contribution in pathogenicity of *C. albicans*, rather a direct role of Dpb3-Dpb4 subcomplex in the virulence of *C. albicans* cannot be ruled out. As the *dpb3/dpb4* null strains are temperature-sensitive, they did not multiply in the host to evade the immune system, leading to infections. To our surprise, mice immunized with the *dpb3*ΔΔ*dpb4*ΔΔ strain did not provide any protection to the fungal lethal re-challenge, probably due to its inability to boost memory immune responses. Thus, not all the avirulent strains can be developed into a whole-cell vaccine. Not surprisingly, aligning with this study, the heat-killed *C. albicans* strain merely protects fungal infections (26). However, the interaction site between Dpb3 and Dpb4 subunits can be explored to develop antifungal drugs. As these subunits share minimum homology with human counterparts, designing specific anti-fungal drugs will be safe and impactful.

**Fig 9:**
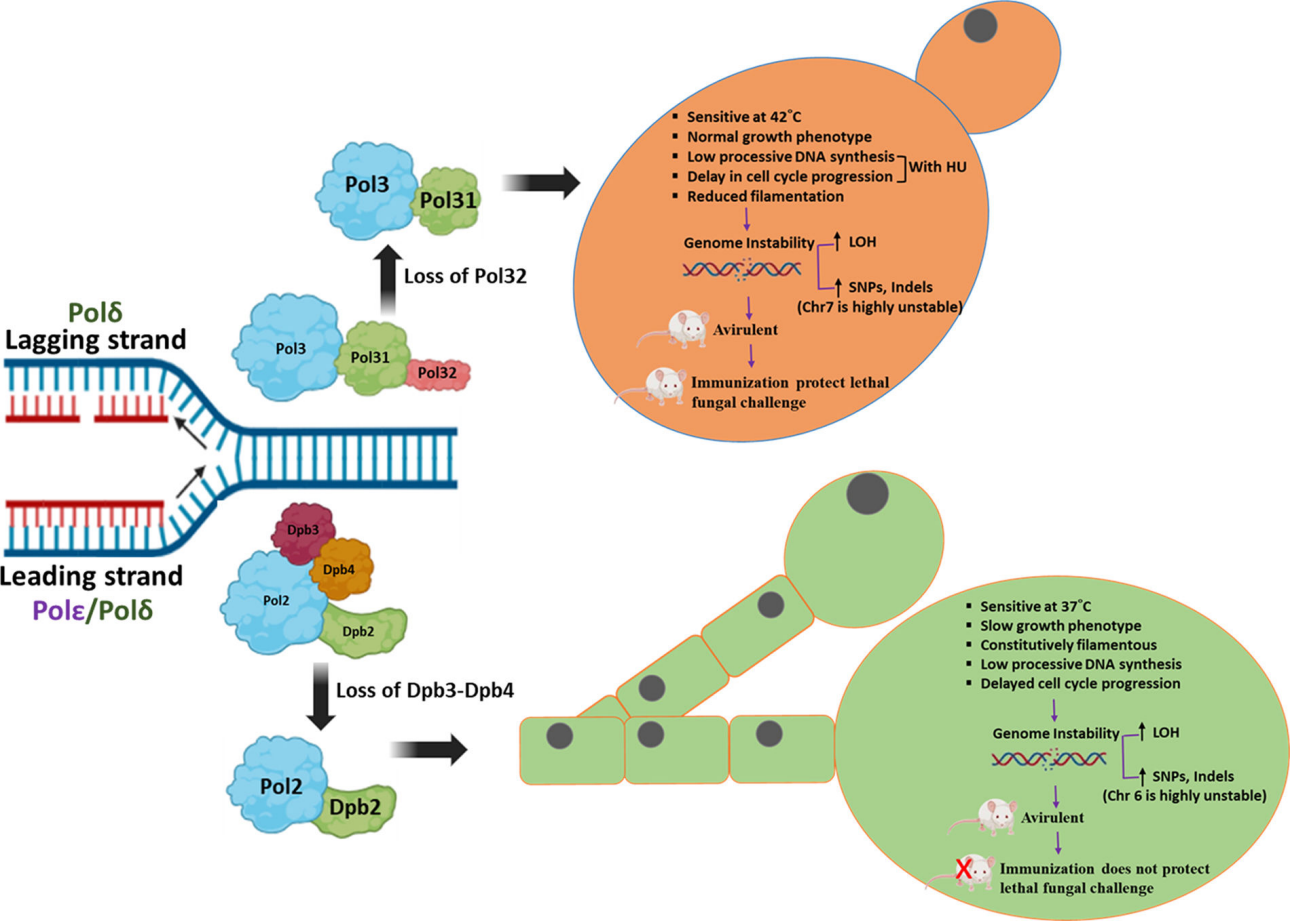
Model showing comparative phenotypes of Polδ- and Polε-defective strains of *C. albicans*. Although Polε majorly participates in leading strand synthesis, Polδ synthesizes both leading and lagging strands during DNA replication. Although their small subunits (Pol32, Dpb3, and Dpb4) are dispensable for growth, all three subunits play important roles in their respective DNA polymerase functions by regulating structural stability, processivity, and fidelity. The model depicts a comparative phenotype exhibited by *C. albicans* strains lacking Pol32 of Polδ and Dpb3 or Dpb4 or both subunits of Polε. They share common phenotypes and also possess distinct phenotypes. Although all these strains are avirulent; only *pol32*ΔΔ as a whole-cell vaccine protects mice against fungal infections. The interacting surfaces of Dpb3 and Dpb4 can be targeted for anti-fungal drug development.

Although Polδ and Polε are involved in synthesizing two opposite strands during DNA replication, the roles of their non-essential subunits Pol32 and Dpb3-Dpb4 subunits, respectively, play very similar roles, and the loss of their subunits in strains exhibits similar phenotypes (31) (Fig. 9). Like *dpb3/dpb4* null strains, *pol32*ΔΔ (CNA25) *C. albicans* cells show sensitivity to HU and DNA-damaging agents, compromised DNA synthesis, enhanced spontaneous mutation rate, antifungal drug resistance, and non-pathogenic in mice model of systemic candidiasis. An Hsp90-dependent mechanism operates in these defective strains to develop anti-fungal drug resistance and robust biofilm formation (31, 54). However, unlike *dpb3/dpb4* null strains that are constitutively filamentous, *pol32*ΔΔ showed reduced filamentation, and being able to multiply at 37°C in the host, it induces immune responses to protect fungal infections as a live whole cell vaccine strain (27). We recommend that temperature-sensitivity phenotype can be considered a critical criterion to identify antifungal drug targets.

In summary, since the loss of Dpb3 and Dpb4 strongly affects cell morphology and viability in mice with consequences on pathogenesis, it appears that while acquiring pathogenicity during evolution, the Dpb3-Dpb4 subcomplex has gained additional functions in regulating fungal morphogenesis and virulence in addition to its role as a structural complex of Polε in DNA replication and genome stability as in non-pathogenic budding yeast. Thus, the interacting interface of Dpb3-Dpb4 subcomplex is a suitable target site for antifungal drug development.

## MATERIALS AND METHODS

### Animal, reagents, and chemicals

In-house breed strains of BALB/c female mice of 6–8 weeks were maintained in individually ventilated cages under the standard condition with *ad libitum*. Oligonucleotides used in this study were procured either from Eurofins Scientific or Integrated DNA Technologies, USA. *C. albicans* (SC5314) and its derivative used in this study are listed in Table 1. The yeast strains were grown in YPD or various synthetic drop-out media as required, and when required, 1% of maltose (#59338) from SRL was added. The restriction enzymes and other enzymes required for cloning were purchased from NEB and Roche, USA. Genotoxins like HU (#102023), Cisplatin (#198872), TBHP (#190049), and MMS (#205518) were procured from MP Biomedicals, whereas nourseothricin was from Jenabioscience. Periodic Acid Schiff (PAS) Stain Kit (#ab150680) was obtained from Abcam, Cambridge, USA, and SYTOX Green (#S7020) was purchased from Thermoscientific.

**TABLE 1:** Various strains used for this study

Strain	Description /genotype	Source
SC5314	*C. albicans* Wild type (WT)	[Manohar et al., (55)]
CNA66	Heterozygous knock out of *DPB3* in SC5314 (*DPB3dpb3*Δ)	This study
CNA115	Homozygous knock out of *DPB3* in SC5314 (*dpb3*ΔΔ)	This study
CNA77	Heterozygous knock out of *DPB4* in SC5314 (*DPB4dpb4*Δ)	This study
CNA130	Homozygous knock out of *DPB4* in SC5314 (*dpb4*ΔΔ)	This study
CNA129	Homozygous knock out of *DPB4* in CNA115 (*dpb3*ΔΔ*dpb4*ΔΔ)	This study
CNA126	Heterozygous knock out of URA3 in SC5314 (*ura3Δ*::*URA3*)	[Manohar et al. (56)]
CNA162	Heterozygous knock out of *URA3* in CNA115 (*dpb3ΔΔura3Δ*::*URA3*)	This study
CNA163	Heterozygous knock out of *URA3* in CNA130 (*dpb4ΔΔura3Δ*::*URA3*)	This study
CNA146	Heterozygous knock out of *URA3* in CNA129 (*dpb3ΔΔdpb4ΔΔura3Δ*::URA3)	This study
CNA179	Reintegration of C-terminal truncation of *DPB4 in* CNA130 *dpb4*ΔΔ::*DPB4*ΔC	This study
CNA180	Reintegration of *DPB4 in* CNA130 *dpb4*ΔΔ::*DPB4*	This study
CNA204	Reintegration of *DPB3 in* CNA115 *dpb3*ΔΔ::*DPB3*	This study
CNA178	Reintegration of C-terminal truncation of *DPB3 in* CNA115 *dpb3*ΔΔ::*DPB3*ΔC	This study
CNA193	Reintegration of N-terminal truncation of *DPB3 in* CNA115 *dpb3*ΔΔ::*DPB3*ΔN	This study
CNA194	Reintegration of C, N-terminal truncation of *DPB3 in*CNA115 *dpb3*ΔΔ::*DPB3*ΔNC	This study
CNA205	Reintegration of CaDpb3 F97A, F101A *in* CNA115 *dpb3*ΔΔ::*DPB3* F97A, F101A	This study
CNA206	Reintegration of CaDpb3 F97A, F101A *in* CNA129 (*dpb3*ΔΔ*dpb4*ΔΔ::*DPB3* F97A, F101A)	This study

### Bioinformatics analyses

The unverified Dpb3 (orf19.306) and Dpb4 (orf19.2088) ORF sequences of *C. albicans* were retrieved from *Candida* Genome Database and aligned similar sequences from *S. cerevisiae*, *S. pombe*, and *Homo sapiens* using T-Coffee tool server. Similarly, H2A and H2B orf sequences were aligned with Dpb3 and Dpb4 sequences of *C. albicans*, respectively. The structural modeling database SWISS-MODEL (https://swisssmodel.expasy.org) was used to generate the model structure of CaDpb3 and CaDpb4, and images were generated by using PyMOL. Dpb3-Dpb4 sub-complex of ScPolε was also retrieved from PDB, and critical interface residues were identified. A conserved region of the interacting interface of CaDpb3-CaDpb4 was identified by sequence alignment.

### Generation of *C. albicans* knock-out and knock-in strains

In order to generate *DPB3* and *DPB4* genes knockout in *C. albicans* SC5314 strain, a modified *SAT1* flipper strategy was deployed. Instead of one deletion cassette, we use two deletion cassettes having different lengths of upstream sequences but having a common downstream sequence for efficient deletion of both alleles of interest (32, 35). The downstream fragments of *DPB3* (241 bp) and *DPB4* (213 bp) genes were PCR amplified by the primer pairs NAP606 (5’- GGCC CCG CGG GAT GAC GTA GTA ATG AAA G-3′)-NAP607 (5’- GGCC GAG CTC CTA AAG ACA TCA CTA G-3′) and NAP611 (5’- GGCC CCG CGG CCA GAC TCT TCA GAA ACC-3′) and NAP612(5′-GGCC GAG CTC GGG CTC AGC AGA TAA AGA G-3′), respectively, and cloned into the SacII-SacI sites of pSFS2. The longer (396 bp) and shorter upstream (310 bp) fragments of *DPB3* gene were amplified by using a common forward primer NAP603 (5’- GGCC GGT ACC CAA CAA GTG GTA G −3’) and separate reverse primers NAP604 (5’- CCGG CTCGAG CTG AGC ATT TCC AAT ATC-3′) and NAP605 (5’- GGCC CTCGAG GTT GAG TTT GTC TAG G-3′). The PCR products were cloned into the KpnI-XhoI sites of the construct containing the downstream sequence to generate pNA1698 and pNA1725, respectively. Similarly, the longer (382 bp) and shorter (325 bp) upstream sequences of *DPB4* were amplified by using NAP608 (5’- GGCC GGT ACC GCG TTT CCG TAC CAT GC −3’) as a common forward and NAP795 (5’- CCGG CTCGA GGA GTT GTT CAA CGC GTC-3′) and NAP610 (5’- CCGG CTCGA GGT GGC ATT GTA TAT TC-3′) as reverse primers separately. Amplified fragments were cloned into the KpnI-XhoI site of a vector containing *DPB4* downstream sequence to generate pNA1726 and pNA1731, respectively. The PCR condition for the amplification of upstream and downstream sequences by using Taq polymerase in a 100 µL reaction included 95°C for 1 min, 95°C for 15 sec, 52°C for 30 sec, and 72°C for 30 sec for 30 cycles. First, by using these constructs, *dpb3*ΔΔ and *dpb4*ΔΔ strains were generated in the SC5314 strain, and again, *dpb4*ΔΔ was generated in the *dpb3*ΔΔ genetic background to obtain a double-gene deletion strain (*dpb3*ΔΔ*dpb4*ΔΔ) by sequentially transforming these deletion cassettes and by recycling the nourseothricin marker by growing the cell in the presence of maltose. We also generated an add-back *dpb3*ΔΔ::*DPB3* strain by integrating the full-length *DPB3* gene into one of the loci in *dpb3*ΔΔ. For reintegration, *DPB3* was amplified using primers NAP603 and NAP910 (5′-GGCC CTC GAG ATA TCT CGA CAC TC-3′) and cloned into KpnI-XhoI sites of pNA1698 to obtain pNA1901construct. The KpnI-SacI fragment from pNA1901 was transformed into the *dpb3*ΔΔ strain, and the transformants were selected on the YPD + NAT plate. The integration of *DPB3* was confirmed by colony PCR with NAP603 and NAP607 primers. Confirm colonies were further grown in YPM media for 3 successive days to cure the NAT cassette, and finally, removal of the cassette was confirmed by the absence of colony on the YPM + NAT agar plate. The NAT cassette was excised by treating the cells with maltose. To strengthen our results, we have also expressed wild-type and mutated Dpb3 orfs under the control of a constitutive *ADH1* promoter. The wild-type and mutant Dpb3 orfs (F97A, F101A) were amplified by using NAP983 (5′-GGCC CCCGGG ATG TCC CAA GAA GAA CAG TC-3′) and NAP984 (5’- GGCC CCCGGG TTT AAT CTT TCA TTA CTA CG-3′) primers, and the obtained PCR product (706 bp) was digested with SmaI and cloned downstream to *ADH1* promote to generate pNA1889. A StuI digested linear fragment from pNA1889 was integrated into the RP10 locus of the *dpb3*ΔΔ strain to obtain the *dpb3*ΔΔ::*ADH1*p-*DPB3* strain. By using a similar approach, Dpb3ΔN (545 bp, NAP982 5′-GGCC CCC GGG ATG CAA GAT GAG TTT CAA AAC AAT CTC-3′- NAP98 5’- GGCC CCC GGG TTT AAT CTT TCA TTA CTA CG-3′), Dpb3ΔC (453 bp, NAP983 5’- GGCC CCCGGG ATG TCC CAA GAA GAA CAG TC-3′ - NAP981 5’- CCGG CCC GGG CTA CTT TTG TTG AAC TAA TTC ACC-3′), and Dpb3ΔNC (293 bp, NAP982 (5’- GGCC CCC GGG ATG CAA GAT GAG TTT CAA AAC AAT CTC-3′- NAP981 5’- CCGG CCC GGG CTA CTT TTG TTG AAC TAA TTC ACC-3′) were amplified and cloned downstream to *ADH1*p construct. The constructs were liberalized by StuI and integrated to generate *dpb3*ΔΔ::*ADH1*p-Dpb3ΔN, *dpb3*ΔΔ::*ADH1*p-Dpb3ΔC, and *dpb3*ΔΔ::*ADH1*p-Dpb3ΔNC strains, respectively. To add back a wild-type and a C-terminal truncated *DPB4*, the upstream sequence fragments were amplified by primer pairs NAP608 (5’- GGCC GGT ACC GCG TTT CCG TAC CAT G-3′) - NAP908 (5’- CCGG CTC GAG CAA TTT CCT AAC TTT CAT TGT CAA G-3′) and NAP608 (5’- GGCC GGT ACC GCG TTT CCG TAC CAT G-3′) - NAP909 (5’- CCGG CTC GAG TCA TCT CTG GTG TTT C-3′), respectively, and cloned into KpnI-XhoI site of construct containing downstream fragment of *DPB4* gene. The KpnI-SacI fragments from these newly generated constructs (pNA1843 and pNA1846) were transformed into *dpb4*ΔΔ to generate *dpb4*ΔΔ*::DPB4* and *dpb4*ΔΔ*::DPB4ΔC* strains.

### Growth curve

Overnight grown cultures of various strains of *C. albicans* were diluted to OD_600_ = 0.1 in 10 mL of YPD broth to achieve an equal number of cells. These strains were allowed to grow further, and absorbance was recorded at an interval of 2 h for 18 h. Experiments were carried out in biological duplicates twice. The graph was plotted using GraphPad Prism 8.0 software.

### Sensitivity assay

The spot-based sensitivity test of various strains of *C. albicans* was carried out as described (57). Briefly, an equal number of fungal cells was serially diluted 10-fold in a 96-well round bottom plate by YPD broth and spotted on YPD plates with or without different mentioned concentrations of genotoxic agents like HU, MMS, Cisplatin, and H2O2. Plates were allowed to grow for 48 h. For UV damage, the completely dried spotted plates were exposed to different dosages of UV radiation and wrapped with aluminum foil. All the plates were allowed to grow for 48 h at 30°C and photographed. For temperature sensitivity, similarly spotted plates were incubated at 16°C, 30°C, 37°C, and 42°C.

### Protein expression constructs and protein purification

CaDpb3 orf was amplified by using a high-fidelity Q5 DNA polymerase from the genomic DNA of SC5314 with the primer pairs NAP11 (5’- GGC CAA GCT TAG ATC TAC ATA TGT CCC AAG AAG AAC AGT CCG G-3′) - NAP12 (5′-GGC CGA ATT CAG ATC TTT AAT CTT TCA TTA CTA CG-3′). The PCR product was digested with BglII and cloned into the BamHI site of pRSETB to generate an N-terminal 6xHis tag in frame with CaDpb3 bacterial expression system. Similarly, CaDpb4 orf PCR product amplified with NAP19 (5′-CCG GCT GCA GGG ATC CAC ATA TGC CAC CAA AAG GTT GGA GAA AAA TGC-3′) - NAP20 (5′-CCG GGG TAC CGG ATC C TT ATTC TTC TTC CTC TTC TTC TTC-3′) primers was digested with BamHI and cloned into same site of pRSETB to generate 6xHis-CaDpb4 bacterial expression construct. To generate CaDpb3 with F97A, F101A mutations, two fragments of the orf, were amplified separately by using primer pairs NAP11 - NAP986 (5′-CT TGC TTG TTC TGC AGC ATA TTG AAC AGC CAA TTC −3’) and NAP985 (5′-GCT ACA GAA TTG GCT GTT CAA TAT GCT GCA GAA CAA G-3′) - NAP12, digested with BglII-PstI and cloned into BamHI site of pRSETB by split-fragment approach. All the constructs were authenticated by DNA sequencing. These constructs were transformed into BL21-DE3 cells, and the protein was overexpressed upon IPTG induction. Briefly, 10 mL of 12–14 h overnight grown pre-culture was added to 1,000 mL of LB broth along with 50 µg/mL of ampicillin and grown at 37°C till the OD_600_ reached 0.6. Then, the culture was induced with 1 mM of IPTG and allowed to grow for an additional 8 h at 30°C. Cells were harvested by centrifugation at 5,000 rpm for 10 min. About 2–3 g of cell pellet was resuspended in 100 mL of lysis buffer (50 mM Tris-HCL pH 7.5, 10 mM imidazole, 400 mM NaCl, 1 mM PMSF, 10% glycerol, and 1X protease inhibitor cocktail (Biopioneer #BPBOI001). Cell suspension was sonicated (50% amplitude, pulse 3 sec on; 5 sec. off, 4°C) for 15 min. The cell debris was discarded by centrifugation at 30,000 rpm for 1 h in an ultracentrifuge. The clear supernatant was incubated with 1 mL His beads for 1 h and then transferred to a column followed by washing with 10 mL buffer containing 20 mM Tris-HCL pH 7.5, 20–40 mM imidazole, 300 mM NaCl, 1 mM PMSF, 1 mM beta-mercaptoethanol for twice. The beads were then washed carefully with a gradual increase in imidazole concentration (100 to 500 mM) in the mentioned wash buffer. The final elution of Dpb3 protein occurs at 300 mM of imidazole and Dpb4 protein at the concentration of 200 mM imidazole. The protein purity and quantification were confirmed after resolving on a 10% SDS-PAGE followed by staining with Coomassie blue. Proteins were stored in small aliquots at −80°C until further use.

### Isothermal calorimetry

The purified WT CaDpb3, CaDpb3 (F97A, F101A), and CaDpb4 proteins were dialyzed overnight in a buffer containing 20 mM HEPES (pH 7.4) and 150 mM NaCl. ITC experiments were performed using a MicroCal PEAQ-ITC system (Malvern Panalytical) at 25°C with a 20 µM concentration of wild-type or mutant CaDpb3 proteins loaded into a sample cell, and 100 µM CaDpb4 was injected into the cell by a given syringe. Twenty-five consecutive injections of 1.5 µL each were made at an interval of 120 s. Heat exchange by a buffer-buffer titration was estimated, and the value was subtracted from the respective individual experiment. Binding isotherms were fitted into a one-site binding model using MicroCal PEAQ-ITC analysis software, and various kinetic parameters were calculated.

### Circular dichroism

The purified proteins were dialyzed with 1 L of dialyzing buffer (20 mM Tris-HCl buffer, pH 7.5, 20 mM NaCl) at 4°C overnight. The secondary structure was determined by circular dichroism (CD) spectroscopy (JASCO 1500). The data acquisition and analysis were done using Spectra Manager software. Spectra were obtained at 25°C in a 10 mm path-length quartz cuvette containing the sample at concentrations of 0.2 mg/mL of protein. The spectra were corrected for the buffer. Mean residue ellipticity values were calculated using the expression [θ] = θ × 100 / (cln), where θ corresponds to ellipticity (in millidegrees), c is the protein concentration (in mol/liter), l is the path length (in cm), and n is the number of amino acid residues. The analysis was repeated twice.

### Pulsed-field gel electrophoresis

Bio-Rad CHEF Yeast Genomic DNA Plug Kit was used to prepare DNA plugs of WT and different knockout strains as per the manufacturer’s instructions. Total chromosomes were isolated from an equal number of overnight-grown cells. The chromosomal DNA separation was performed in 1.2% agarose gel with 0.5 × TBE buffer using a CHEF Mapper XA system. The running condition was voltage 6v × cm^−1^, switch time 60 to 120 s, angle 120° for 36 h followed by voltage 4.5 v × cm^−1^, switch time 120 s to 300 s, angle 120° for another 12 h. The buffer was changed every 12-h interval. The gel was then stained with ethidium bromide overnight, and the image was captured in a Gel Doc system.

### Alkali agarose gel electrophoresis

Cells from overnight cultures of *C. albicans* strains were diluted in 100 mL fresh YPD media to get an OD_600_ = 1 and allowed further to grow for 2 h at 30°C. One hundred millimolar hydroxyurea was then added and allowed to grow for another 45 min at 30°C. Cells were harvested by centrifugation at 5,000 RPM for 5 min. The cell pellets were washed twice with autoclaved distilled water to remove HU, followed by resuspension in a fresh 100 mL of YPD broth and growing. Cells were collected at different time periods of growth (0, 6, 12, and 24 h) to determine DNA post-damage recovery. Total genomic DNA was isolated and quantified using a nanodrop and confirmed by resolving it in a 1% agarose gel. Subsequently, an equal amount of DNA isolated from various strains at different recovery periods was subjected to resolve in alkaline agarose gel as described previously (56). Gel was neutralized and stained with EtBr, and the image was captured in a Gel doc system. Band intensity was estimated by Image J.

### Mutagenesis assay

The rate of spontaneous mutation in different knockout strains in comparison to WT strain was measured as described before (35). For that, heterozygous *ura3Δ/URA3* strains were generated in the *dpb3*ΔΔ, *dpb4*ΔΔ, *dpb3*ΔΔ*dpb4*ΔΔ, *dpb3*ΔΔ::*DPB3*, and WT strains. A similar method as described previously for gene knockout was used to delete a single copy of the *URA3* gene. The upstream and downstream flanking regions of *URA3* were amplified using primer pairs NAP746 (5’- CCG GGG TAC CGT CAT TCC TCT TG-3′) - NAP747 (5′-GGC CCT CGA GAC TGG TGA GGC ATG AG −3’) and NAP748 (5′-GGC CCC GCG GGA TGC TGG TTG GAA TG −3’) - NAP749 (5′-CCG GGA GCT CGA AGA TTA TAA TGA TGT TC −3’) from the genomic DNA and cloned into KpnI-XhoI sites and SacII- SacI sites of pSFS2, respectively, to generate a deletion cassette containing construct pNA1760. KpnI–SacI gene deletion cassette was transformed into various *C. albicans* strains, and the heterozygous deletion was confirmed by PCR using NAP746 and NAP749 primers. To determine the rate of mutagenesis, the strains were grown in the YPD medium at 30°C overnight. The cells were then harvested, washed twice with sterile distilled water, and diluted to get an appropriate number of cells. Around 50–200 cells were spread on SD agar plates without or with 1 mg/mL FOA. All the plates were incubated at 30°C for 3–4 days. The FOA-resistant colonies of each strain were counted for SD and SD + FOA agar plates. The complete loss of *URA3* in these strains in the presence of 5FOA was verified by growing them on media lacking uracil. The experiments were performed in biological quadruplicates with technical duplicates.

### Cell cycle analysis

Cell cycle progression assay was performed by staining the cellular DNA using SYTOX green as described earlier (37). Briefly, a single colony of freshly streaked WT and *dpb3*∆∆*dpb4*∆∆ strains was inoculated in 5 mL YPD broth and grown at 30°C for 16 h to get synchronized populations. After that, cells were re-inoculated in 10 mL YPD broth with a final OD_600_ = 0.3, allowed to grow, and cells were harvested with a regular interval of 30 min up to 3 h. Harvested cells were fixed, and RNA and proteins were degraded by respective enzyme treatments overnight. The permeable cells were stained with SYTOX green and transferred to FACS tubes for acquisition in the LSR BD Fortessa Flow cytometer. Data were analyzed in Flowjo software. *C. albicans* cells were distinguished from debris by plotting SSC-A versus FSC-A, followed by singlet discrimination using FSC-H versus FSC-A, and finally, singlet cells were visualized by blue laser excitation (488 nm). Analyzed data were exported in the JPEG format.

### Expression of phosphorylated Rad53

Overnight grown cells of WT and *dpb3*ΔΔ*dpb4*ΔΔ strains were diluted in 100 mL fresh YPD media to an OD_600_ = 0.5 and allowed to grow for another 2 h at 30°C. Furthermore, one set was treated with 50 mM HU for 2 h at 30°C, whereas the other set remained untreated. Cells were harvested by centrifugation at 5 K RPM for 5 min. In order to prepare the cell lysate, 200 µL of homogenizing buffer (50 mM Tris HCl pH 7.5, NaCl 150 mM, DOC 0.5%, EDTA 0.5 mM, NP40 1%, and PMSF 1 mM) supplemented with 1× protease inhibitor and 200 µL of 0.5 mm glass bead were added and subjected to homogenization. Debris was cleared by centrifugation at 12 K RPM for 10 min, and supernatant was collected. Equal amounts of cell lysates were resolved on 10% SDS-PAGE. Gel was transferred to 0.45 µm PVDF membrane by running at 90V for 2 h. Blocking was done with 5% skim milk in 1× TBST buffer for 2 h. Blot was gently rinsed to remove excessive skim milk, whereas the upper portion of the blot (75–135 kDa region) was incubated with anti-Rad53 antibody (Abcam #4874) with a dilution of 1:1,000 with 1× TBST overnight, the lower portion of the blot (20–50 kDa region) was probed with self-raised anti-PCNA antibody. After that, blots were washed thrice with 1xTBST for 5 min each. Then, an anti-rabbit secondary antibody (1:10,000) conjugated to HRP was added, which was diluted in 2.5% skim milk and incubated for 1 h at room temperature. After incubation, the blot was again washed five times for 5 min each. Bands were visualized with WesternBLoT Chemiluminescence HRP substrate ECL (TaKaRa) on the ChemiDoc Imaging System (Biorad).

### Whole-genome sequencing

Total genomic DNA was isolated from WT and *dpb3*ΔΔ*dpb4*ΔΔ stain, and DNA libraries were generated based on the microbial whole-genome sequencing recommendations of the NexteraTM DNA flex library preparation Kit from Illumina Inc. The purified DNA libraries were quantified using a QubitR fluorometer, and the average library size was determined by loading an appropriate dilution on a D1000 screen tape. After QC clearance, about 4 nM libraries were loaded on a MiSeq v3 kit, and sequencing was conducted for 2 × 150 cycles. The genome analysis was carried out as described before (31). Briefly, the FASTQ files (containing paired-end reads sequences) were processed for adapter-free reads and trimmed using Trimmomatic. Shovill was used for *de novo* assembly of filtered data. The reads were aligned with the reference genome assembly of *C. albicans* SC5314 (ASM18296v3). SNPs and Indels were filtered out by using Variant calling. To get the variant with high confidence, the standard hard filtering recommendation of GATK (https://gatk.broadinstitute.org/hc/en-us/articles/360035890471-Hard-filteringgermlineshortvariants) was applied. SnpEff database was used for variant annotation, and VCFPolyXutility of Jvarkit was used to determine repeated reference bases around the variant position.

### Gene expression analysis

MagSureTM all RNA Isolation kit (#MAR-100) was used to isolate RNA from the overnight grown cultures of WT and *dpb3*ΔΔ*dpb4*ΔΔ strain. For cDNA preparation, the Applied Biosystems kit (# 4368814) was used. qRT- PCR was performed using 5 µL of 10 times diluted cDNA sample. The virulence associated genes (*ALS3, ECE1, SAP3, SAP6, EFG1, CPH1, NRG1, TUP1,* and *HWP1*) expression was recorded taking GAPDH as control. The primers used are given in Table 2. The 20 µL reaction mixture for qRT-PCR included 100 ng of cDNA, 10 pm of primer mix, and 10 µL of SYBR green master mix (Applied Biosystems, #A25742). PCR cycle conditions included 95°C for 2 min, 95°C for 30 sec, and 60°C for 30 sec for 35 cycles. Experiments were carried out in biological duplicates along with technical triplicates. The ΔΔct values were calculated, and fold change values of *dpb3*ΔΔ*dpb4*ΔΔ compared with wild type were plotted using GraphPad Prism 8 software. The semi-quantitative PCR of the mentioned genes was performed in a 20 µL reaction with 100 ng of cDNA, 10 pico moles primer mix for each gene, 200 µM dNTPs, 1× Taq buffer, and 1U of Taq DNA polymerase (Sigma). The PCR conditions were 95°C for 1 min, 95°C for 30 sec, and 60°C for 30 sec for 30 cycles. The amplified PCR products were analyzed on a 1% agarose gel, and the gel image was captured using a Chemi XRS Gel Documentation system.

**TABLE 2:** Primers used for real-time PCR

Gene name	Forward primer	Reverse primer
*GAPDH*	5’-gaccgttgacggtccatcc-3	5’-catcggtggttgggactc-3’
*ALS1*	5’-cctatctgactaagactgcacc-3’	5’-acagttggatttggcagtgga-3’
*ALS3*	5’-cggttgcgactgcaaagac-3’	5’-gaccaacccaaaacagcattcc-3’
*HWP1*	5’-cagttccactcatgcaaccatc-3’	5’-gcaataccaataatagcagcaccg-3’
*ECE1*	5’-ccggcatctcttttaactgg-3’	5’-gagatggcgttccagatgtt-3’
*SAP3*	5’-gttactggtccccaaggtg-3’	5’-cttgtccttgaccagcttgac-3’
*SAP6*	5’-gtcaacgctggtgtcctc-3’	5’-gcaggaacggagatcttgag-3’
*NRG1*	5’-cacctcacttgcaacccc-3’	5’-gccctggagatggtctga-3’
*TUP1*	5’-ctcttggcgacaggtgcag-3’	5’-gtggtgacgccgtcttcga-3’

### Morphology of *C. albicans*

Overnight grown *C. albicans* cells were diluted to an OD_600_ = 1 with 5 mL YPD medium and grown further at 30°C for 1 h, and the cell morphology was captured under a Leica microscope with 40× magnification. The % of various forms of cells was quantified. Multiple focuses were taken into account to count about 100 cells on a single slide. Mean values for three independent experiments were represented.

### Mouse model of systemic candidiasis and fungal burden analyses

Female BALB/c mice (*n* = 6) of 6–8 weeks were intravenously administered with 5 × 10^5^ CFU of WT, *dpb3*∆∆, *dpb3*∆∆::*DPB3*, *dpb3*∆∆*dpb4*∆∆ strains along with 100 µL saline control. Mice survivability was monitored for 30 days. The fungal inoculum was prepared by pelleting down the overnight grown culture in YPD + chloramphenicol (34 mg/mL) broth by centrifugation at 10 K RPM for 1 min, followed by thoroughly washing in autoclaved water and normal saline, and then, cells were re-suspended in normal saline. Cells were counted manually by using a Neubauer chamber slide in the central area (5 × 5 squares), 5 × 10^6^ cells/mL cell suspension was prepared, and only 100 µL was injected. The cell count was also re-verified by a plating assay. The survival curve was plotted using GraphPad Prism 8 software. An autopsy was done, and vital organs were collected. Although one kidney was used for CFU determination, the other one was stored in formalin for tissue sectioning and PAS staining. Fungal burden was also estimated in spleen and liver tissues by CFU analysis on a YPD + chloramphenicol agar (34 mg/mL) plate. The survived mice beyond 30 days were allowed to survive till their natural death.

For the antifungal protection assay, immunized BALB/c mice (*n* = 6/group) with *dpb3*∆∆ and *dpb3*∆∆*dpb4*∆∆ strains were generated as mentioned above, and after 30 days of survival, they were re-challenged intravenously with 5 × 10^5^ CFU WT *C*. *albicans* (2°), and mice survivability was monitored. As control experiments, similar aged group naïve mice were injected with WT cells and saline. The mice that succumbed or were sacrificed as per the standard humane endpoints were dissected, and vital organs were collected to confirm the death caused due to *C. albicans* infections by CFU and PAS staining analyses. The survival curve was plotted using GraphPad Prism 8 software for each group of mice.

### Statistical analysis

Statistical significance of the growth curve, qRT-PCR, and hyphal cell counts were calculated based on two-way analysis of variance (ANOVA) using Dunnett’s multiple comparison test and Sidak’s multiple comparison test. respectively. For the spontaneous mutagenesis assay, one-way analysis of variance (ANOVA) using Tukey’s multiple-comparison test was used. Statistical significance in all Kaplan-Meier survival graphs was determined using the log-rank (Mantel-Cox) test. Significance levels were indicated by *P* values as * ≤0.05, ** ≤0.01, and *** ≤0.001.

## Data Availability

The data that support the findings of this study are available from the corresponding author upon reasonable request. The whole genome sequence data of *dpb3*ΔΔ*dpb4*ΔΔ and WT SC5314 strains can be retrieved from the NCBI database using the accession numbers PRJNA1058882 and PRJNA862296.

## SUPPLEMENTAL MATERIAL

Supplemental figures mbio.01227-24-s0001.pdf Figures S1 to S5.

Supplemental information mbio.01227-24-s0002.docx Legends for supplemental figures; Tables S1 to S3; caption for Table S4.

Table S4 mbio.01227-24-s0003.xlsx Number of homozygous indels in the repeat regions of chromosomes of *dpb3*ΔΔ*dpb4*ΔΔ strain of *C. albicans*.

ASM does not own the copyrights to Supplemental Material that may be linked to, or accessed through, an article. The authors have granted ASM a non-exclusive, world-wide license to publish the Supplemental Material files. Please contact the corresponding author directly for reuse.
