# Supplementary figures and images for: Critical roles of Dpb3-Dpb4 sub-complex of DNA polymerase epsilon in DNA replication, genome stability, and pathogenesis of Candida albicans
Source: mBio. 2024 Aug 29;15(10):e01227-24. doi: 10.1128/mbio.01227-24 (PMC11481497; doi:10.1128/mbio.01227-24)

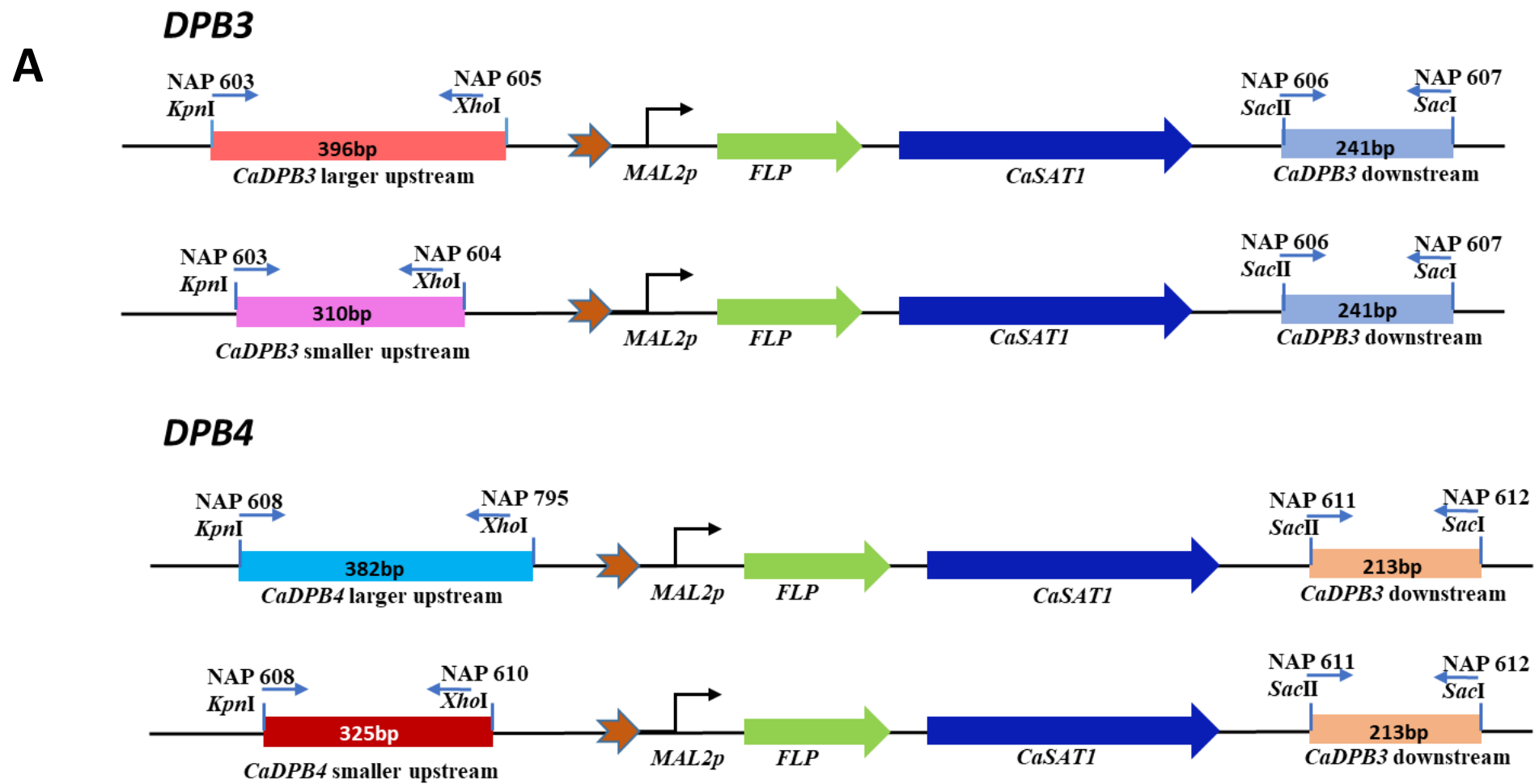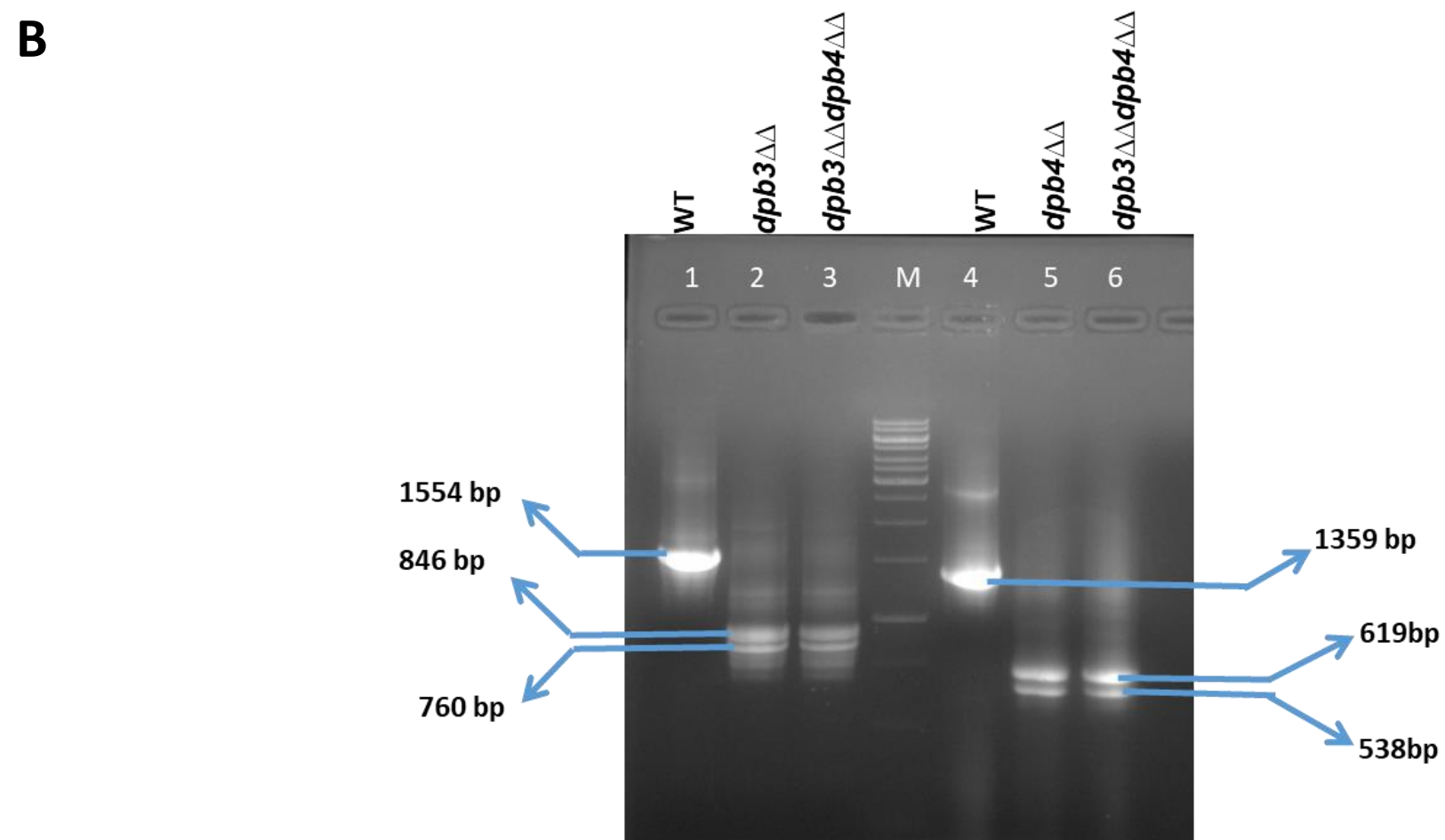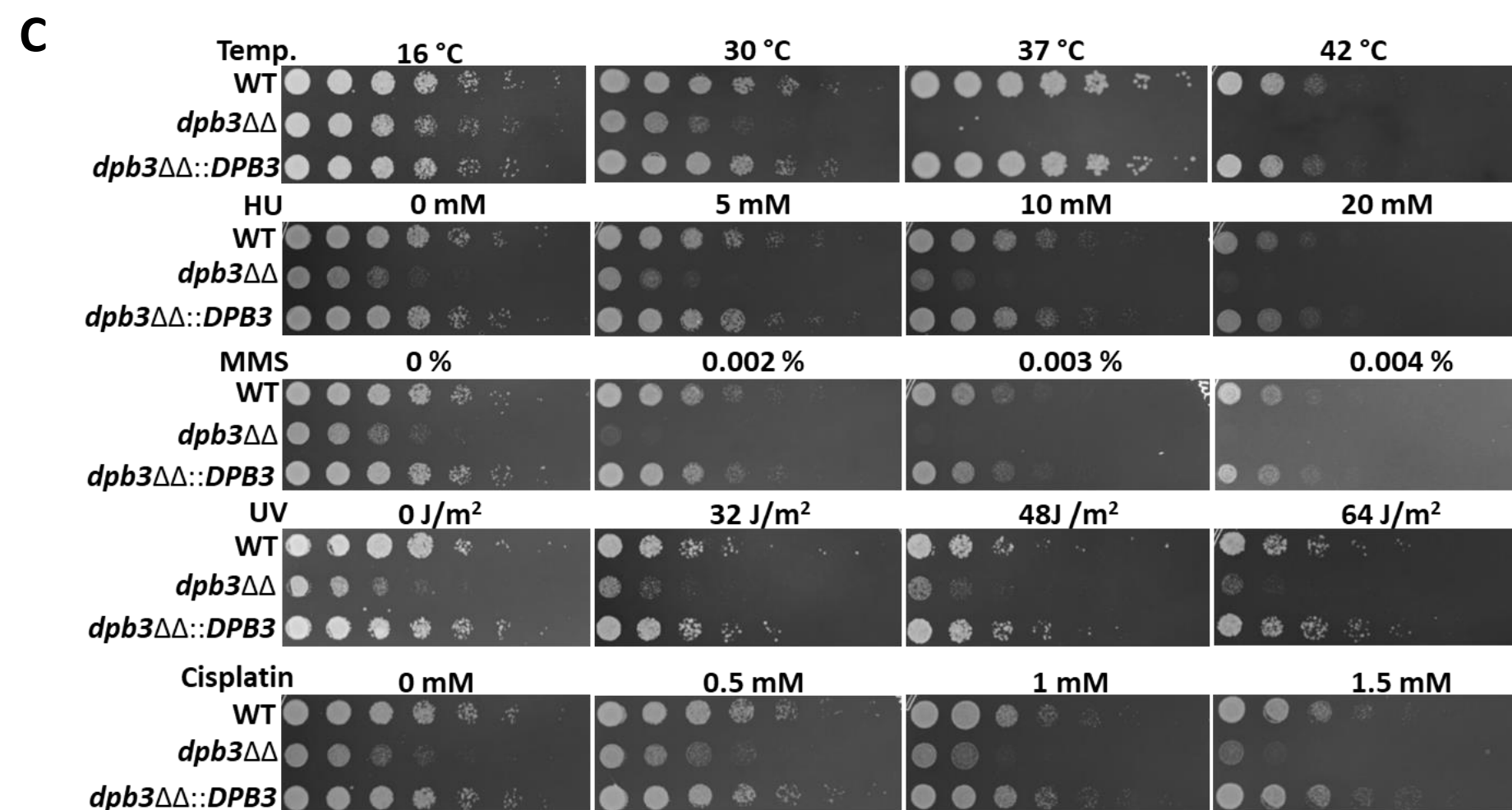

Figure S2

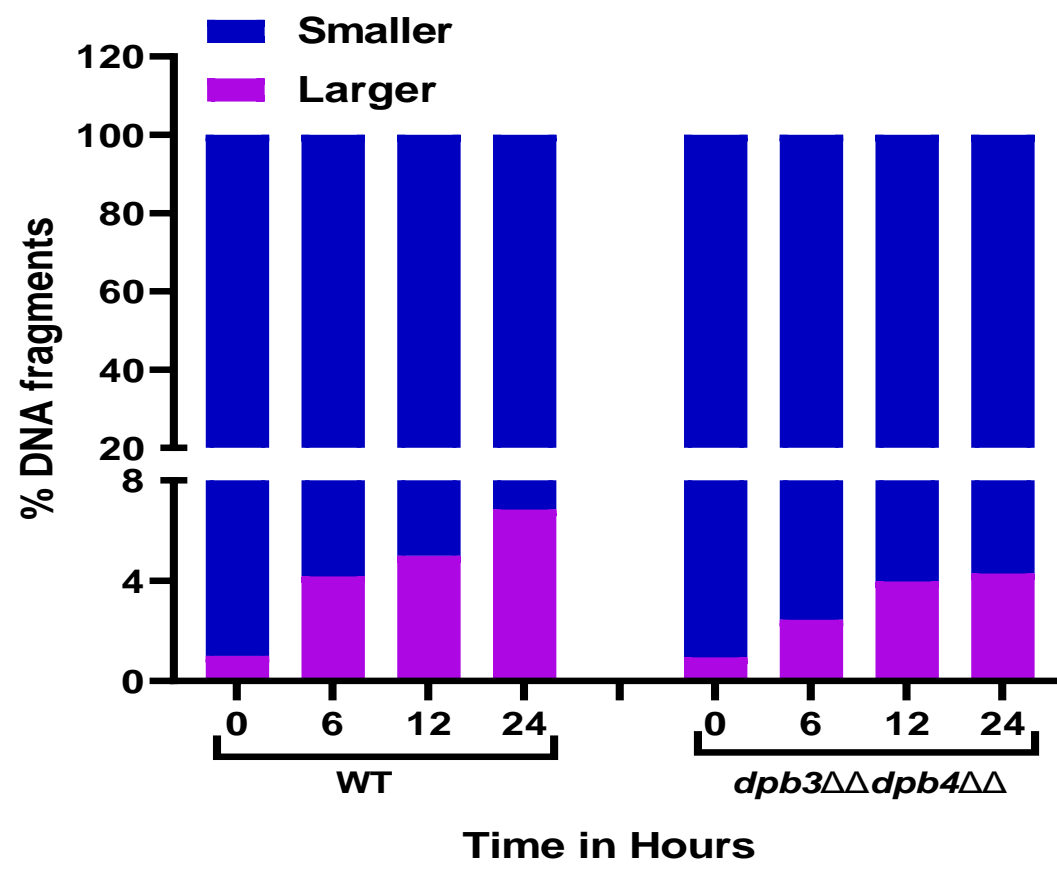

Figure S3

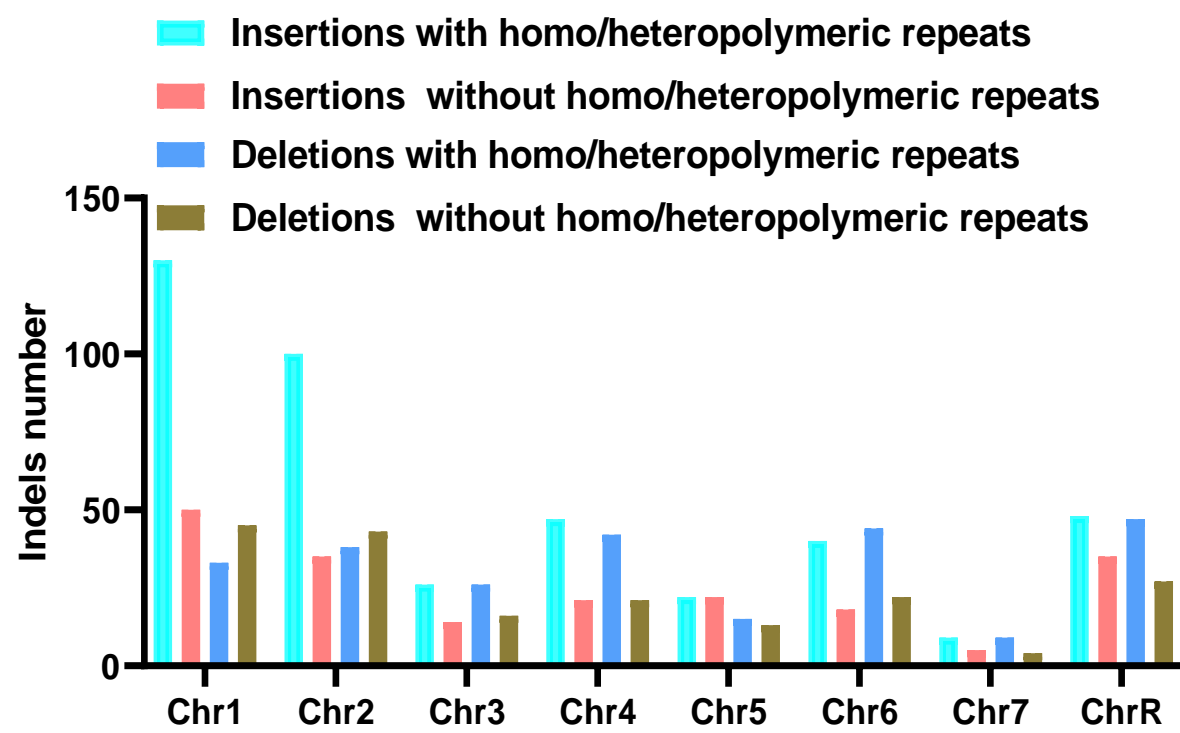

Figure S4

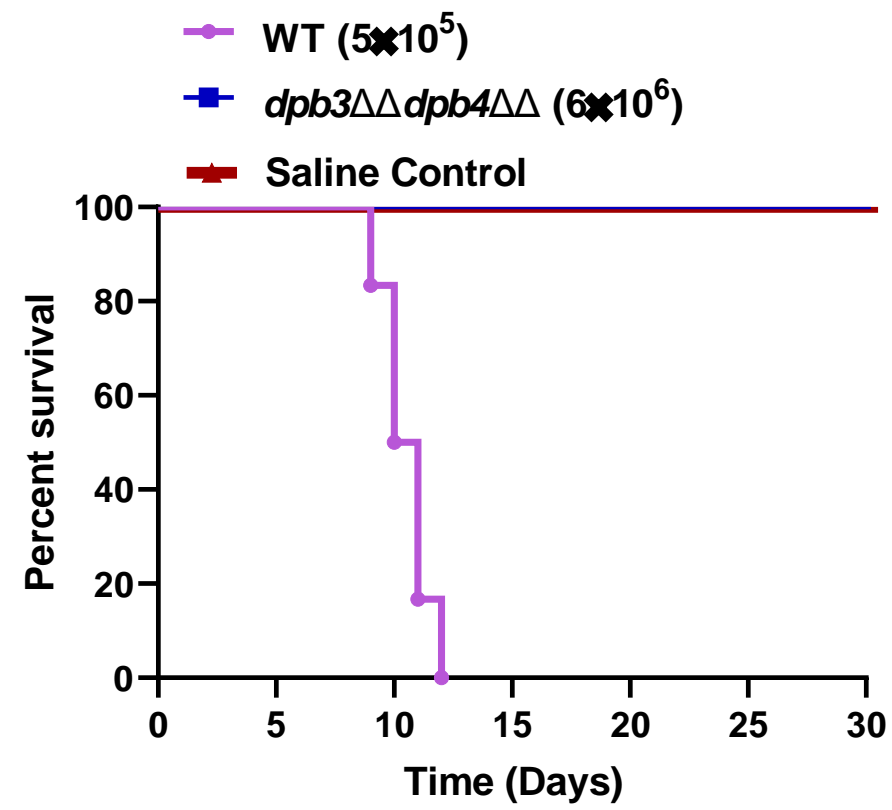

Figure S5

Supplement: Supplemental figures — Figures S1 to S5. [file mbio.01227-24-s0001.pdf]
