# Supplementary material for: Critical roles of Dpb3-Dpb4 sub-complex of DNA polymerase epsilon in DNA replication, genome stability, and pathogenesis of Candida albicans
Source: mBio. 2024 Aug 29;15(10):e01227-24. doi: 10.1128/mbio.01227-24 (PMC11481497; doi:10.1128/mbio.01227-24)
Supplement: Supplemental information — Legends for supplemental figures; Tables S1 to S3; caption for Table S4. [file mbio.01227-24-s0002.docx]

**Supplementary Information**

**Critical roles of Dpb3-Dpb4 sub-complex of DNA polymerase epsilon in DNA replication, genome stability, and pathogenesis of *Candida albicans***

Bhabasha Gyanadeep Utkalaja^1,2^, Shraddheya Kumar Patel^1,2^, Satya Ranjan Sahu^1,2^, Abinash Dutta^1^, and Narottam Acharya^1,^*

^1^Laboratory of Genomic Instability and Diseases, Department of Infectious Disease Biology, Institute of Life Sciences, Bhubaneswar-751023, India.

^2^Regional center of Biotechnology, Faridabad, India.

***Correspondence to:**

Narottam Acharya, Phone: 91-674-2304278, Fax: 91-674-230 0728

E-mail: narottam_acharya@ils.res.in; [narottam74@gmail.com](mailto:narottam74@gmail.com)

Running title: Essential role of Dpb3-Dpb4 subunits

Key words: *Candida albicans*, DNA replication, DNA polymerase, whole genome sequencing, Systemic candidiasis, virulence

**Figure Legends:**

**Figure S1: Amino acid sequence alignment of CaDpb3 with CaH2A and CaDpb4 with CaH2B.** Amino acids of Dpb3 and Dpb4 with H2A and H2B of *C. albicans* were aligned using T-Coffee tool server, respectively. The conserved histone fold (HF) in both the proteins was highlighted. Identical residues were indicated with * symbols and distantly similar residues were indicated with ; and . symbols.

**Figure S2: Generation of *DPB3* and *DPB4* gene knockouts in *C. albicans*.** Ray diagrams of deletion constructs containing upstream and downstream fragments of *DPB3* and *DPB4* genes of *C. albicans* flanking the *SAT1* selection re-cycling marker was shown. Various primer positions were as indicated (**A**). Diagnostic PCR confirmed the homozygous deletion of *DPB3* and *DPB4* genes in various knockout strains (**B**). Spot assay of WT, *dpb3*ΔΔ and *dpb3*ΔΔ::*DPB3 C. albicans* strains was carried out by spotting various dilutions of the pre-culture on YPD agar plate without or with different concentrations of HU, MMS, and cisplatin. For UV treatment, spotted plates were subjected to different times of UV-B exposure. Plates were allowed to grow for 48 hrs at 30^°^C and photographed. For temperature sensitivity, spotted plates were incubated at 16^°^C, 30^°^C, 37^°^C, and 42^°^C. Spots were allowed to grow for 48 hrs and then photographed (**C**).

**Figure S3: Role CaDpb3/CaDpb4 in DNA replication.** The percentage of smaller (blue) and larger (purple) DNA fragments accumulation in wild type and *dpb3*ΔΔ*dpb4*ΔΔ cells after recovery post HU treatment in alkaline gel electrophoresis and ethidium bromide staining analysis were determined.

**Figure S4: Indels in the repeat regions of the genome of *dpb3*ΔΔ*dpb4*ΔΔ strain.** The number of insertions and deletions in the homo- and hetero-repeat regions of various chromosomes were plotted. Repeat regions vary from 1 to 24 nucleotide repeats in the wild type genome.

**Figure S5: Animal protection assay:** BALB/c mice (n=6) were immunized with 6x10^6^ CFUs of *dpb3*ΔΔ*dpb4*ΔΔ strains (1°). After 30 days, mice were re-challenged with WT (2°). As control experiments six each mice were challenged with WT and saline and survival was monitored. Graph was plotted using graph pad prism 8 software.

**Table S1: Percentage of identity and similarity of Dpb3 and Dpb4 with other proteins**

| Proteins | Percent Identity | Percent Similarity |
| --- | --- | --- |
| CaDpb3/ScDpb3 | 17.72% | 31.8% |
| CaDpb3/SpDpb3 | 13% | 20.2% |
| CaDpb3/HsDpb3 | 12.9% | 24.6% |
| CaDpb3/CaH2A | 11.3% | 23.8% |
| CaDpb4/ScDpb4 | 31.6% | 47.5% |
| CaDpb4/SpDpb4 | 26.8% | 43.9% |
| CaDpb4/HsDpb4 | 9.8% | 17.1% |
| CaDpb4/CaH2B | 10.7% | 20.6% |

**Table S2: Number of indels (insertion and deletion) in various chromosomes of *dpb3*ΔΔ*dpb4*ΔΔ strain of *C. albicans***

**
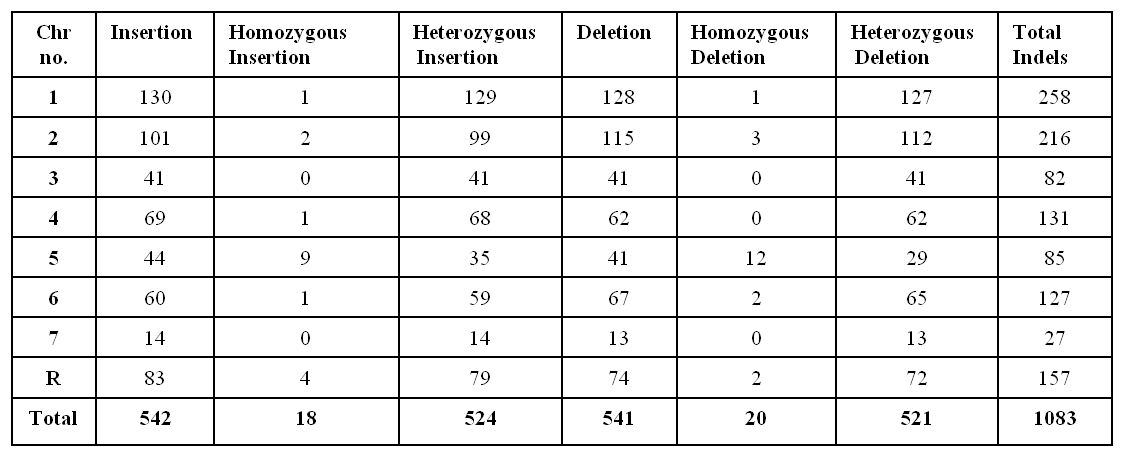

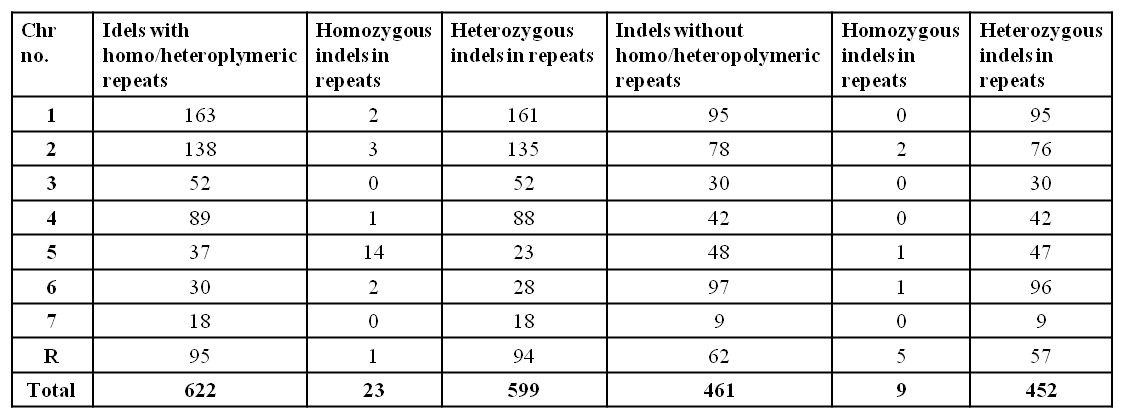
**

**Table S3: Number of SNPs in various chromosomes of *dpb3*ΔΔ*dpb4*ΔΔ strain of *C. albicans***

**
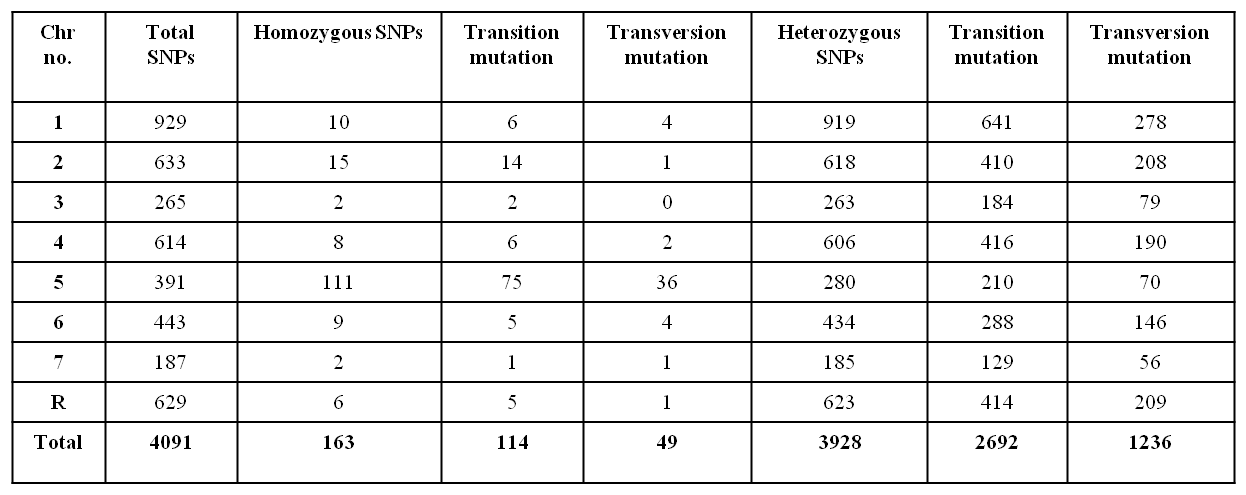

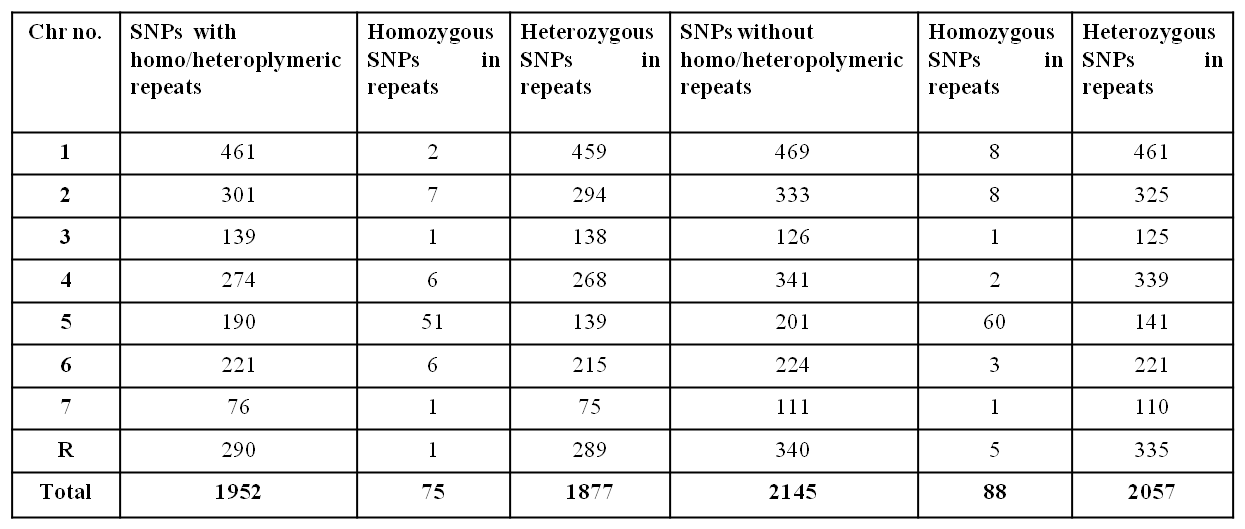
**

**Table S4: Number of homozygous indels in the repeat regions of chromosomes of *dpb3*ΔΔ*dpb4*ΔΔ strain of *C. albicans***
